# Association between *FCGR2A* rs1801274 and *MUC5B* rs35705950 variations and pneumonia susceptibility

**DOI:** 10.1186/s12881-020-01005-1

**Published:** 2020-04-06

**Authors:** Xueshu Shi, Yue Ma, Haiyan Li, Huanxin Yu

**Affiliations:** 1 Nursing Division, The second affiliated Hospital of Tianjin University of Traditional Chinese Medicine, Tianjin, 300150 P.R. China; 2 Endoscopic Skull Base Surgery Center, Tianjin Huanhu Hospital, No 6, JiZhao Road, Jinnan District, Tianjin, 300350 P.R. China; 3 Department of Otorhinolaryngology Head and Neck Surgery, Tianjin Huanhu Hospital, Tianjin, 300350 P.R. China

**Keywords:** *FCGR2A*, *MUC5B*, Pneumonia, Susceptibility, Variation

## Abstract

**Background:**

Herein, we collected currently published data to comprehensively evaluate the impact of the *FCGR2A* (Fc fragment of IgG receptor IIa) rs1801274 and *MUC5B* (mucin 5B, oligomeric mucus/gel-forming) rs35705950 variations on susceptibility to pneumonia diseases.

**Methods:**

We retrieved case-control studies from three online databases and applied the statistical approach of meta-analysis for a series of pooling analyses.

**Results:**

A total of fourteen case-control studies were included for *FCGR2A* rs1801274; while thirty-one case-control studies were included for *MUC5B* rs35705950. No significant difference between pneumonia cases and controls for *FCGR2A* rs1801274 was found. However, *MUC5B* rs35705950 was significantly associated with pneumonia susceptibility in the whole population under the genetic models of allelic T vs. G [OR (odds ratio) =3.78], carrier T vs. G (OR = 3.31), TT vs. GG (OR = 13.66), GT vs. GG (OR = 4.78), GT + TT vs. GG (OR = 5.05), and TT vs. GG + GT (OR = 6.47) (all *P* < 0.001, Bonferroni-adjusted *P* < 0.006; false discovery rate-adjusted *P* < 0.0010). Furthermore, we observed a similar positive result for subgroup analyses of “Caucasian”, “Asian”, “population-based control”, and “idiopathic pulmonary fibrosis”.

**Conclusions:**

*MUC5B* rs35705950, but not *FCGR2A* rs1801274, increases susceptibility to clinical pneumonia, especially to idiopathic pulmonary fibrosis, in both the Caucasian and Asian populations.

## Background

Pneumonia is a group of viral or bacterial infection-induced lung disorders that can cause the symptoms of fever, cough, shortness of breath and fatigue [1–3]. There are various types of pneumonia, such as idiopathic pulmonary fibrosis (IPF), nonspecific interstitial pneumonia (NSIP), idiopathic interstitial pneumonia (IIP) [4–6]. Although the pathogenesis of pneumonia remains elusive, environmental exposure factors (e.g., tobacco smoking, virus or bacterial infection) and genetic variants may contribute to the susceptibility to pneumonia [2, 7, 8]. Here, we investigated pneumonia-associated gene variations. After database retrieval and publication selection, we excluded reports of potential pneumonia-associated gene variation without enough or updated data, and finally focused on two variants, namely, *FCGR2A* rs1801274 and *MUC5B* rs35705950.

The human *FCGR2A* gene in the 1q23 chromosome region encodes a member of the heterogeneous Fc fragment of the IgG receptor family of immune receptors and contains a functional rs1801274 variation in exon 4, which leads to the amino acid alteration from histidine (H) to arginine (R) at position 131 of the FCGR2A protein [9, 10]. No prior meta-analysis regarding the genetic role of *FCGR2A* rs1801274 in pneumonia susceptibility has been reported, and studies have reported different findings [11–20]. Therefore, this is the first pooling analysis that has been conducted to assess the potential association between *FCGR2A* rs1801274 and overall pneumonia risk and was carried out according to PRISMA (preferred reporting items for systematic reviews and meta-analyses) guidelines.

Human *MUC5B*, a gel-forming mucin gene in the 11p15.5 chromosome region [21], can be expressed by certain bronchial epithelial cells and contains several variations (such as rs35705950) in a three kilobase region upstream of the *MUC5B* transcription start site [22, 23]. We found one meta-analysis published in 2013 [24] and two meta-analyses published in 2015 [25, 26] regarding the association between *MUC5B* rs35705950 and the risk of idiopathic pulmonary fibrosis. However, only ten studies were included, and the genetic association between *MUC5B* rs35705950 and the susceptibility to other pneumonia types has not yet been investigated. Therefore, we performed an updated meta-analysis to comprehensively assess the effect of *MUC5B* rs35705950 on the risk of overall pneumonia disease based on available case-control studies as of February 2020.

## Methods

### Database search and study identification

Referring to similar investigations [27, 28], we collected potentially relevant studies from three databases, including PubMed, EMBASE (Excerpta Medica Database), and WOS (Web of Science), on 25 February 2020. To prevent the filtering of the possible eligible studies, in the retrieval strategy, we utilized a combination of subject words [“MeSH (medical subject headings)” for PubMed and “Emtree” for EMBASE] and free words (“Entry Terms” for PubMed, “synonyms” for EMBASE) in the retrieval strategy. No restriction on publication language or region was applied. The detailed search terms can be found in Additional file 1: Table S1.

Based on our exclusion/inclusion criteria, we then performed the study identification and eligibility assessment. The exclusion criteria were as follows: (i) meta-analysis, comment or review; (ii) mice or cell data; (iii) conference abstract or case reports; (iv) other gene or disease; (v) lack of normal controls or specific data; and (vi) not in line with HWE (Hardy-Weinberg equilibrium) principle. Only those studies with allelic or genotypic frequency data from both the pneumonia cases and negative controls were enrolled.

### Evidence collection and quality appraisal

After full-text verification of each case-control study, we extracted and sorted the basic information (e.g., the first name of the author, publication date, race, variation, frequency) in Excel files. We also contacted corresponding authors by email in an attempt to retrieve any missing data of allelic or genotypic frequency. We also applied the NOS (Newcastle-Ottawa quality assessment scale) system to evaluate the study quality.

### Statistical approaches

To assess the statistical heterogeneity between studies, we performed I^2^/Q statistical tests by means of the STATA software (version 12.0 Stata Corporation, USA). The DerSimonian-Laird random effects model was used for high heterogeneity [I^2^ > 50% or *P*_heterogeneity_ (*P* value in the heterogeneity test) < 0.05]; while the Mantel-Haenszel method for fixed-effect models was used for low or no heterogeneity (I^2^ < 50% and *P*_heterogeneity_ > 0.05). We also utilized the STATA software to conduct association tests and obtained the OR, 95% confidence interval (CI) and *P*_*association*_ (*P* value in the association test) under a total of six models to assess the association strength.

Referencing the relevant literature [29, 30], we also adjusted the *P* value in the association tests using the p.adjust function of the R software, version 3.6.1. *P* values adjusted for the Bonferroni and false discovery rate (FDR) were also obtained. Furthermore, we performed a false-positive report probability (FPRP) analysis to assess the probability of a true genetic relationship using the genetic analysis “gap” R package [31, 32] [FPRP cutoff value = 0.2, power OR = 1.5, and prior probability levels = (0.25, 0.1, 0.01, 0.001, 0.0001, 0.00001)].

### Trial sequential analysis

Referencing the previous studies [33, 34], we carried out the trial sequential analysis (TSA) test for the assessment of conclusion robustness using the TSA viewer software (http://www.ctu.dk/tsa/) (type I error probability = 5%, statistical test power = 80%, and relative risk reduction =20%).

### Publication bias and sensitivity analyses

Additionally, we conducted a group of subgroup analyses by the factors of race, control source, and pneumonia type. We also carried out the Begg and Egger tests for analyzing the presence of publication bias, and the sensitivity analysis was conducted using one-by-one removal of each study to evaluate data stability.

## Results

### Eligible case-control study characteristics

**Fig. 1:**
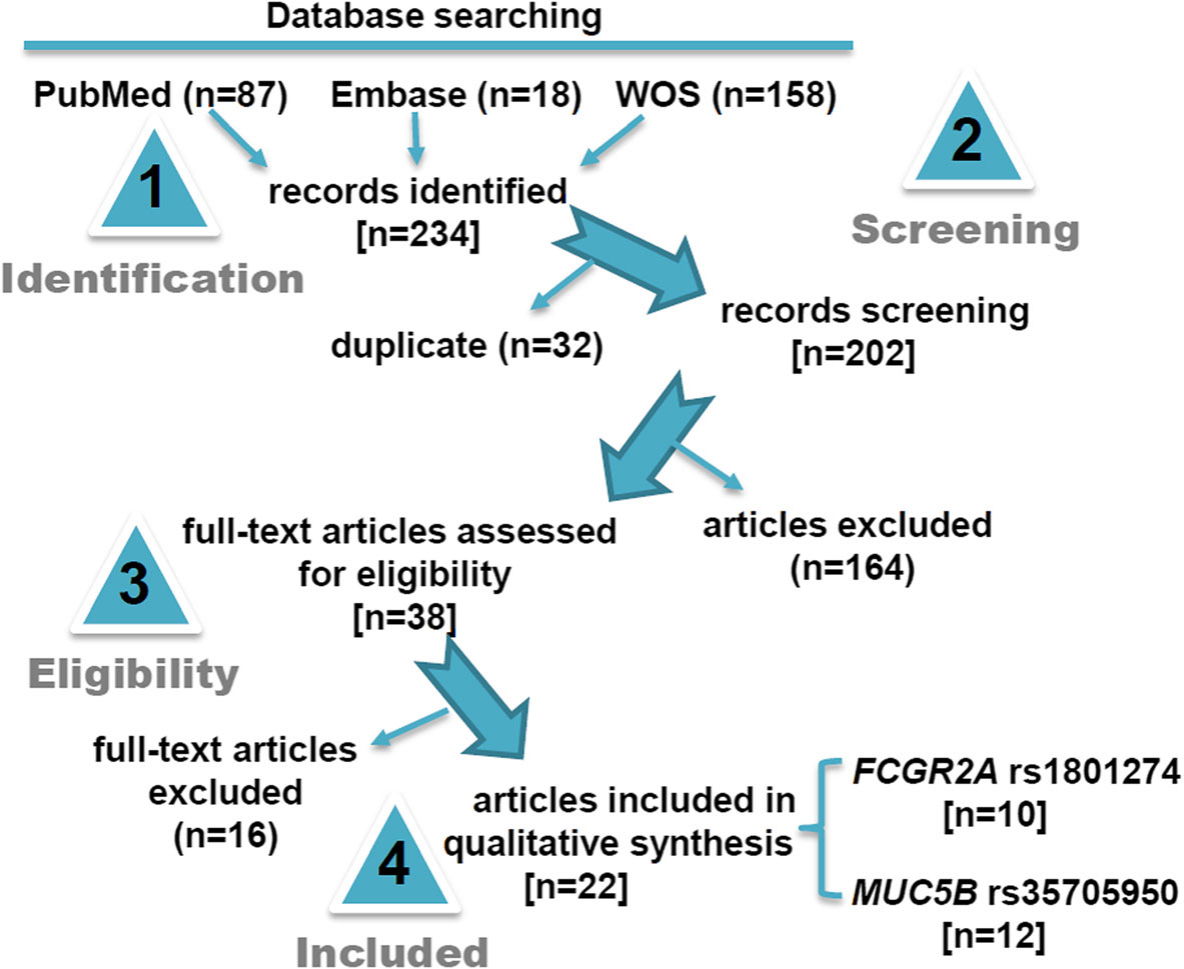
Flow diagram of eligible case-control study selection

Figure 1 shows the flow diagram of selecting eligible case-control studies. Utilizing the computerized database search process, we collected a total of 234 records (PubMed 87 records, EMBASE 18 records, and WOS 158 records). We then removed 32 duplicate records, as well as 164 records due to the following exclusion criteria: meta, comment or review (*n* = 101), mice or cell (*n* = 17), conference abstract or case report (*n* = 9), other gene or disease (*n* = 37). Another 16 records were excluded due to “lack of normal control or specific data” or “not in line with HWE”. Finally, a total of twenty-two articles [8, 11–20, 23, 35–44] were included in the quantitative synthesis. Fourteen case-control studies from ten articles [11–20] were included in the meta-analysis of *FCGR2A* rs1801274; while thirty-one case-control studies from twelve articles [8, 23, 35–44] were included for *MUC5B* rs35705950. Table 1 presents a summary of the basic information, while Additional file 1: Table S2 presents the detailed allelic and genotypic frequency data of the enrolled case-control studies. All studies were of high quality (all NOS score > =5), as shown in Additional file 1: Table S3.

**Table 1:** Basic information of the studies included in the meta-analysis

First author, Year [Ref.]	Race	Variation	Disease type	Sample		Control source	Assay
				case	control		
Bougle, 2012 [11]	Caucasian	rs1801274	ICU /pneummonia	198	2789	HB	Taqman
Dressen, 2018 [35]	Caucasian	rs35705950	IPF	1510	1874	HB	Sequencing
Endeman, 2009 [12]	Caucasian	rs1801274	CAP	200	313	PB	Taqman
Forthal, 2007 [13]	Mixed	rs1801274	AIDS/ pneumonia	80	478	HB	Allele-specific PCR
Horimasu, 2015 [36]	Asian	rs35705950	NSIP	30	310	PB	Taqman
			IPF	44	310	PB	Taqman
	Caucasian	rs35705950	NSIP	31	35	PB	Taqman
			IPF	71	35	PB	Taqman
Johnson, 2017 [37]	Caucasian	rs35705950	IIP	60	134	NA	Taqman
Jonsson, 2006 [14]	Caucasian	rs1801274	C2D/pneumonia	30	200	PB	Allele-specific PCR/sequencing
Kinder, 2007 [15]	Mixed	rs1801274	SLE/pneumonia	42	217	HB	Pyrosequencing
Kishore, 2016 [38]	Caucasian	rs35705950	IPF^a^	41	96	PB	Sequenom MassARRAY
		rs35705950	IPF^b^	33	96	PB	Sequenom MassARRAY
		rs35705950	IPF^c^	36	96	PB	Sequenom MassARRAY
		rs35705950	IPF^d^	51	96	PB	Sequenom MassARRAY
Ley, 2017 [39]	Mixed	rs35705950	CHP^e^	123	503	PB	Taqman
			CHP^f^	65	503	PB	Taqman
			IPF^e^	147	503	PB	Taqman
			IPF^f^	126	503	PB	Taqman
Moens, 2006 [16]	Caucasian	rs1801274	pneumonia	55	100	Mixed	allelic discrimination
Noth, 2013 [8]	Mixed	rs35705950	IPF^g^	542	542	PB	Genome-Wide Human SNP array
			IPF^h^	544	687	PB	iPLEX Gold Platform
			IPF^i^	324	702	PB	iPLEX Gold Platform
Seibold, 2011 [23]	Mixed	rs35705950	IPF	492	322	PB	Sequenom iPLEX
			FIP	83	322	PB	Sequenom iPLEX
Sole, 2011 [17]	Caucasian	rs1801274	pneumococcal CAP	319	1224	Mixed	ASRED
			nonbacteremic CAP	234	1224	Mixed	ASRED
			bacteremic CAP	85	1224	Mixed	ASRED
Stock, 2013 [40]	Caucasian	rs35705950	IPF	110	416	PB	Taqman
van, 2016 [41]	Caucasian	rs35705950	spIPF	115	249	PB	Taqman
			FIP	55	249	PB	Taqman
			iNSIP	43	249	PB	Taqman
			CTD-IP	35	249	PB	Taqman
Wang, 2014 [42]	Asian	rs35705950	IPF	165	1013	PB	PCR-RFLP/Taqman/ sequencing
			CTD-NSIP	191	1013	PB	PCR-RFLP/Taqman/sequencing
			CTD-UIP	49	1013	PB	PCR-RFLP/Taqman/sequencing
			pneumonia	103	1013	PB	PCR-RFLP/Taqman/sequencing
Wei, 2014 [43]	Caucasian	rs35705950	IPF	84	689	PB	Taqman
Yee, 2000 [18]	Mixed	rs1801274	bacteremic pneumonia	42	136	HB	quantitative flow cytometry
			nonbacteremic pneumonia	28	136	HB	quantitative flow cytometry
Yuan, 2003 [19]	Caucasian	rs1801274	pneumonia	63	20	PB^#^	SSP
			pneumonia	63	58	PB^%^	SSP
Zhang, 2011 [44]	Caucasian	rs35705950	IPF^j^	246	166	PB	Taqman
			IPF^k^	95	636	PB	Taqman
Zuniga, 2012 [20]	Mixed	rs1801274	pneumonia	91	98	PB	Taqman

*Ref.* reference, *M* major allele (A for rs1801274; G for rs35705950), *m* minor allele (G for rs1801274; T for rs35705950), *ICU* intensive care unit, *IPF* idiopathic pulmonary fibrosis, *CAP* community-acquired pneumonia, *AIDS* acquired immune deficiency syndrome, *NSIP* nonspecific interstitial pneumonia, *IIP* idiopathic interstitial pneumonia, *C2D*, homozygous C2 deficiency, *SLE* systemic lupus erythematosus, *CHP* chronic hypersensitivity pneumonitis, *FIP* familial interstitial pneumonia, *spIPF* sporadic type of idiopathic pulmonary fibrosis, *iNSIP* idiopathic non-specific interstitial pneumonia, *CTD-IP* connective tissue disease associated intersititial pneumonia, *CTD-NSIP* connective tissue diseases-nonspecific interstitial pneumonia pattern, *CTD-UIP* connective tissue diseases-usual interstitial pneumonia pattern, *HB* hospital-based, *PB* population-based, NA not available, *ASRED* allele-specific restriction enzyme digestion, *PCR* polymerase chain reaction, *SNP* single nucleotide polymorphism*, SSP* sequence specific PCR, *RFLP* restriction fragment length polymorphism

*a* data of Czech Republic, *b* data of Germany, *c* data of Greece, *d* data of France,

*e* data of University of California San Francisco, *f* data of University of Texas Southwestern,

*g* stage one of genome-wide association study, *h* stage two of genome-wide association study,

*i* stage three of genome-wide association study, *j* data of University of Pittsburgh, k, data of University of Chicago,

*#* healthy control, *%* normal random blood donors

### *FCGR2A* rs1801274

**Fig. 2:**
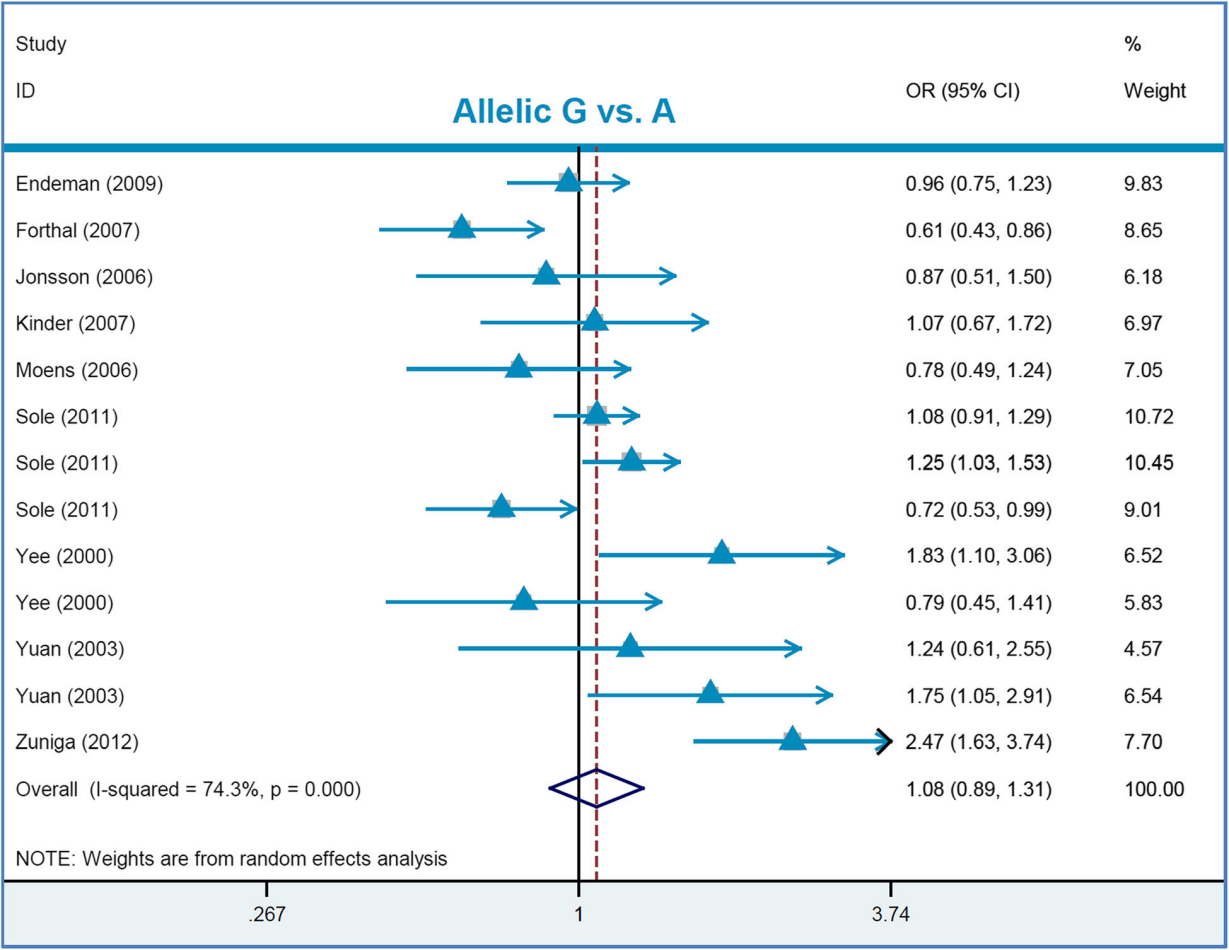
Forest plot of *FCGR2A* rs1801274 under the allelic model

As presented in Table 2, the lack of high inter-study heterogeneity (I^2^ = 26.0%, *P*_heterogeneity_ = 0.182) resulted in using a fixed-effect model for the carrier G vs. A model, whereas a random-effect model was applied for the others. Upon the pooling analysis of thirteen studies (1332 cases /5428 controls) of the overall population, we failed to observe a significant difference between pneumonia cases and negative controls under the allelic G vs. A, carrier G vs. A, GG vs. AA, AG vs. AA, AG + GG vs. AA, or GG vs. AA+AG models (Table 3, all *P*_association_ > 0.05; Bonferroni-*P*_association_ > 0.05; FDR-*P*_association_ > 0.05). Next, we conducted a series of subgroup analyses by race or control source under the six genetic models. As shown in Table 3, we observed similar negative results in all subgroups (all *P*_association_ > 0.05, Bonferroni-*P*_association_ > 0.05; FDR-*P*_association_ > 0.05), except in the subgroup analysis of “HB” under the AG vs. AA model (*P*_association_ = 0.023, Bonferroni-*P*_association_ = 0.138; FDR-*P*_association_ = 0.1380). Fig. 2 shows the forest plot data under the allelic model (as an example). These findings showed that *FCGR2A* rs1801274 may not be strongly associated with overall pneumonia susceptibility.

**Table 2:** Data of heterogeneity assessment and publication bias analysis

Gene (Variation)	Genetic model	Heterogeneity		Fixed/Random	Publication bias	
		I^2^	*P*_*heterogeneity*_		*P*_*Begg*_	*P*_*Egger*_
***FCGR2A*(rs1801274)**	Allelic G vs. A	74.3%	< 0.001	Random	0.760	0.882
	Carrier G vs. A	26.0%	0.182	Fixed	0.310	0.830
	GG vs. AA	71.9%	< 0.001	Random	1.000	0.899
	AG vs. AA	62.3%	0.001	Random	0.428	0.337
	AG + GG vs. AA	68.7%	< 0.001	Random	0.855	0.872
	GG vs. AA+AG	64.3%	0.001	Random	0.443	0.395
***MUC5B*(rs35705950)**	Allelic T vs. G	72.4%	< 0.001	Random	0.683	0.382
	Carrier T vs. G	0.7%	0.449	Fixed	0.740	0.182
	TT vs GG	0.0%	0.870	Fixed	0.488	0.070
	GT vs. GG	59.4%	< 0.001	Random	0.174	0.002
	GT + TT vs. GG	63.4%	< 0.001	Random	0.194	0.002
	TT vs. GG + GT	0.0%	0.959	Fixed	0.373	0.477

*P*_*heterogeneity*_*P* value in the heterogeneity test, *P*_*Begg*_*P* value in the Begg test, *P*_*Egger*_*P* value in the Egger test

**Table 3:** Pooling data regarding the association between *FCGR2A* rs1801274 and pneumonia risk

Genetic model	Subgroup	Sample size		Association			
		Study	Case/control	OR (95% CI)	*P*_*association*_	Bonferroni-*P*_*association*_	FDR-*P*_*association*_
**Allelic G vs. A**	Overall	13	1332/5428	1.08 [0.89,1.31]	0.450	1.000	0.6312
	Caucasian	8	1049/4363	1.03 [0.87,1.22]	0.742	1.000	0.8904
	PB	5	447/689	1.35 [0.88,2.08]	0.169	1.000	0.3015
	HB	4	192/967	0.97 [0.60,1.58]	0.906	1.000	0.9060
**Carrier G vs. A**	Overall	13	1332/5428	1.05 [0.94,1.16]	0.378	1.000	0.6312
	Caucasian	8	1049/4363	1.04 [0.92,1.16]	0.554	1.000	0.8904
	PB	5	447/689	1.16 [0.95,1.43]	0.153	0.918	0.3015
	HB	4	192/967	0.93 [0.71,1.22]	0.603	1.000	0.8364
**GG vs. AA**	Overall	13	1332/5428	1.13 [0.78,1.64]	0.526	1.000	0.6312
	Caucasian	8	1049/4363	1.06 [0.76,1.48]	0.729	1.000	0.8904
	PB	5	447/689	1.76 [0.74,4.19]	0.201	1.000	0.3015
	HB	4	192/967	0.86 [0.40,1.84]	0.697	1.000	0.8364
**AG vs. AA**	Overall	13	1332/5428	0.87 [0.65,1.16]	0.341	1.000	0.6312
	Caucasian	8	1049/4363	0.94 [0.71,1.25]	0.684	1.000	0.8904
	PB	5	447/689	1.04 [0.63,1.73]	0.879	1.000	0.8790
	HB	4	192/967	0.55 [0.32,0.92]	0.023	0.138	0.1380
**AG + GG vs. AA**	Overall	13	1332/5428	0.96 [0.72,1.30]	0.812	1.000	0.8120
	Caucasian	8	1049/4363	0.98 [0.74,1.30]	0.899	1.000	0.8990
	PB	5	447/689	1.27 [0.70,2.30]	0.430	1.000	0.5160
	HB	4	192/967	0.67 [0.42,1.09]	0.106	0.636	0.3180
**GG vs. AA + AG**	Overall	14	1530/8217	1.19 [0.93,1.52]	0.178	1.000	0.6312
	Caucasian	9	1247/7152	1.05 [0.85,1.29]	0.662	1.000	0.8904
	PB	5	447/689	1.71 [0.94,3.13]	0.081	0.486	0.3015
	HB	5	390/3756	1.12 [0.67,1.87]	0.669	1.000	0.8364

*PB* population-based, *HB* hospital-based, *OR* odds ratio, *CI* 95% confidence interval, *P*_*association*_*P* value in the association test, *Bonferroni*-*P*_association_ Bonferroni-adjusted *P* value in the association test, *FDR*-*P*_association_ false discovery rate-adjusted *P* value in the association test

### *MUC5B* rs35705950

**Fig. 3:**
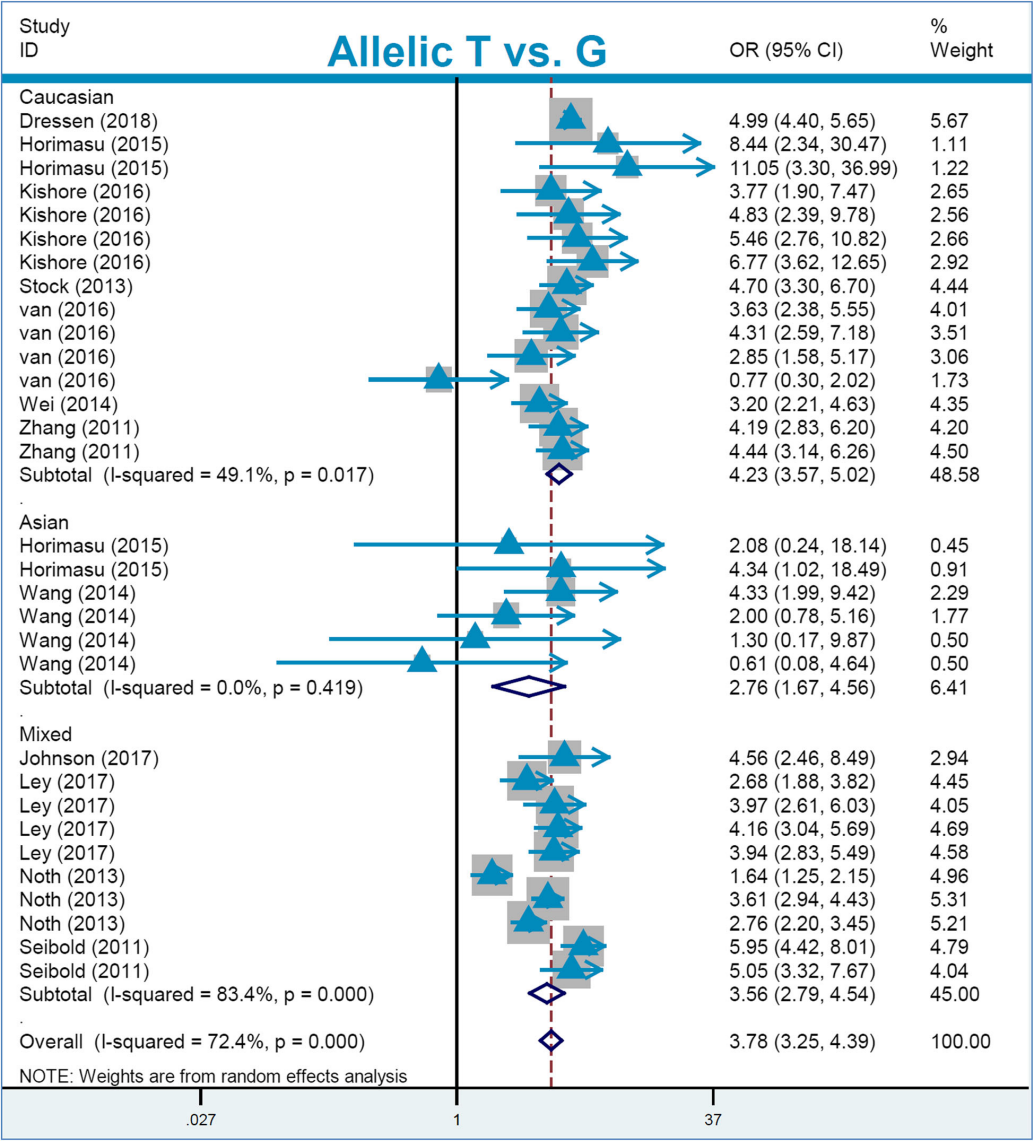
Subgroup analysis of “race” for *MUC5B* rs35705950 under the allelic model

**Fig. 4:**
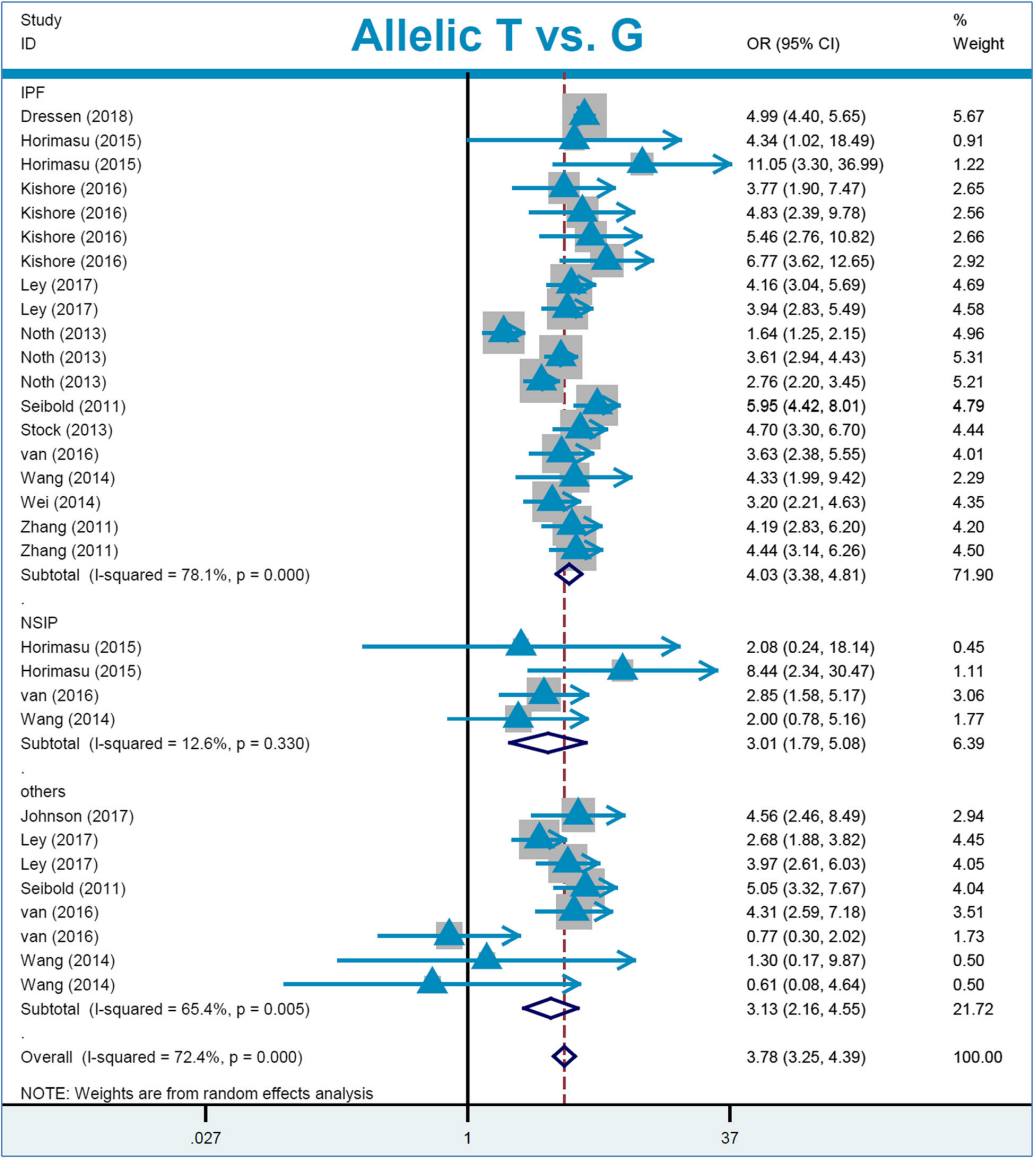
Subgroup analysis of “pneumonia type” for *MUC5B* rs35705950 under the allelic model

For the pooling analysis of *MUC5B* rs35705950, we included a total of thirty-one studies with 5644 cases and 14,624 controls (Table 4) in the overall population. The lack of high inter-study heterogeneity led to using a fixed-effect model for the carrier G vs. A model (Table 2, I^2^ = 0.7%, *P*_heterogeneity_ = 0.449), TT vs. GG (I^2^ = 0.0%, *P*_heterogeneity_ = 0.870), and TT vs. GG + GT (I^2^ = 0.0%, *P*_heterogeneity_ = 0.959), but a random-effect model was used for the others. The pooling results in Table 4 show a significant association between *MUC5B* rs35705950 and high susceptibility to pneumonia in the overall population under the genetic models of allelic T vs. G (OR = 3.78), carrier T vs. G (OR = 3.31), TT vs. GG (OR = 13.66), GT vs. GG (OR = 4.78), GT + TT vs. GG (OR = 5.05), and TT vs. GG + GT (OR = 6.47) (all *P*_association_ < 0.001, Bonferroni-*P*_association_ < 0.006; FDR-*P*_association_ < 0.0010). Subsequently, we observed similar positive correlations in the followed subgroup analyses of “Caucasian”, “PB”, and “IPF” under all genetic models (Table 4, OR > 3, *P*_association_ < 0.01, Bonferroni-*P*_association_ < 0.006; FDR-*P*_association_ < 0.0010). In the “NSIP” subgroup, we detected positive results under the allelic (Table 4, OR = 3.01), carrier (OR = 2.66), and GT + TT vs. GG (OR = 3.19) (*P*_association_ < 0.001, Bonferroni-*P*_association_ < 0.006; FDR-*P*_association_ < 0.0015). In the “Asian” subgroup, we also observed a positive association between *MUC5B* rs35705950 and the high risk of pneumonia under the genetic models of allelic T vs. G (OR = 2.76), carrier T vs. G (OR = 2.47), GT vs. GG (OR = 2.78), and GT + TT vs. GG (OR = 2.78) (all *P*_association_ < 0.001, Bonferroni-*P*_association_ < 0.006; FDR-*P*_association_ < 0.0010). Furthermore, based on the applicable conditions of FPRP [31, 32], we applied FPRP analysis for the data of the “Asian” subgroup. As shown in Additional file 1: Table S4, the observed FPRP values under the prior probability level of 0.1 were all less than 0.20, confirming notable associations. Forest plots of the subgroup analyses are presented in Figs. 3 and 4 and Additional file 2: Figures S1-S6. The above evidence demonstrated that *MUC5B* rs35705950 is closely linked to a high susceptibility to pneumonia diseases, especially to idiopathic pulmonary fibrosis, in the Asian and Caucasian populations.

**Table 4:** Pooling data regarding the association between *MUC5B* rs35705950 and pneumonia risk

Genetic model	Subgroup	Sample size		Association			
		Study	Case/control	OR (95% CI)	*P*_*association*_	Bonferroni-*P*_*association*_	FDR-*P*_*association*_
**Allelic T vs. G**	Overall	31	5644/14,624	3.78 [3.25,4.39]	< 0.001	< 0.006	< 0.0010
	Caucasian	15	2556/5231	4.23 [3.57,5.02]	< 0.001	< 0.006	< 0.0010
	Asian	6	582/4672	2.76 [1.67,4.56]	< 0.001	< 0.006	< 0.0010
	PB	29	4074/12,616	3.69 [3.15,4.32]	< 0.001	< 0.006	< 0.0010
	IPF	19	4776/9031	4.03 [3.38,4.81]	< 0.001	< 0.006	< 0.0010
	NSIP	4	295/1607	3.01 [1.79,5.08]	< 0.001	< 0.006	< 0.0015
**Carrier T vs. G**	Overall	21	3138/9903	3.31 [3.01,3.65]	< 0.001	< 0.006	< 0.0010
	Caucasian	15	2556/5231	3.35 [3.03,3.70]	< 0.001	< 0.006	< 0.0010
	Asian	6	582/4672	2.47 [1.52,4.02]	< 0.001	< 0.006	< 0.0010
	PB	20	1628/8029	3.10 [2.69,3.57]	< 0.001	< 0.006	< 0.0010
	IPF	13	2601/5772	3.43 [3.10,3.80]	< 0.001	< 0.006	< 0.0010
	NSIP	4	295/1607	2.66 [1.66,4.26]	< 0.001	< 0.006	< 0.0015
**TT vs. GG**	Overall	15	2556/5231	13.66 [10.01,18.63]	< 0.001	< 0.006	< 0.0010
	Caucasian	15	2556/5231	12.66 [10.01,18.63]	< 0.001	< 0.006	< 0.0010
	PB	14	1046/3357	10.45 [6.66,16.37]	< 0.001	< 0.006	< 0.0010
	IPF	11	2392/4449	13.87 [10.08,19.09]	< 0.001	< 0.006	< 0.0010
	NSIP	2	74/284	12.29 [1.61,93.86]	0.016	< 0.096	< 0.0015
**GT vs. GG**	Overall	21	3138/9903	4.78 [3.76,6.06]	< 0.001	< 0.006	< 0.0010
	Caucasian	15	2556/5231	5.33 [4.20,6.77]	< 0.001	< 0.006	< 0.0010
	Asian	6	582/4672	2.78 [1.66,4.65]	< 0.001	< 0.006	< 0.0010
	PB	20	1628/8029	4.56 [3.60,5.78]	< 0.001	< 0.006	< 0.0010
	IPF	13	2601/5772	6.35 [5.49,7.34]	< 0.001	< 0.006	< 0.0010
	NSIP	4	295/1607	2.91 [1.72,4.92]	0.010	0.060	0.0120
**GT + TT vs. GG**	Overall	21	3138/9903	5.05 [3.96,6.45]	< 0.001	< 0.006	< 0.0010
	Caucasian	15	2556/5231	5.70 [4.46,7.27]	< 0.001	< 0.006	< 0.0010
	Asian	6	582/4672	2.78 [1.66,4.65]	< 0.001	< 0.006	< 0.0010
	PB	20	1628/8029	4.82 [3.78,6.16]	< 0.001	< 0.006	< 0.0010
	IPF	13	2601/5772	6.49 [5.48,7.68]	< 0.001	< 0.006	< 0.0010
	NSIP	4	295/1607	3.19 [1.75,5.83]	< 0.001	< 0.006	< 0.0015
**TT vs. GG + GT**	Overall	15	2556/5231	6.47 [4.77,8.77]	< 0.001	< 0.006	< 0.0010
	Caucasian	15	2556/5231	6.47 [4.77,8.77]	< 0.001	< 0.006	< 0.0010
	PB	14	1046/3357	5.76 [3.76,8.85]	< 0.001	< 0.006	< 0.0010
	IPF	11	2392/4449	6.45 [4.72,8.81]	< 0.001	< 0.006	< 0.0010
	NSIP	2	74/284	8.41 [1.11,63.80]	0.040	0.240	0.0400

*PB* population-based, *IPF* idiopathic pulmonary fibrosis, *NSIP* nonspecific interstitial pneumonia, *OR* odds ratio, *CI* 95% confidence interval, *P*_*association*_*P* value in the association test, *Bonferroni*-*P*_association_ Bonferroni-adjusted *P* value in the association test, *FDR*-*P*_association_ false discovery rate adjusted-*P* value in the association test

### Publication bias

**Fig. 5:**
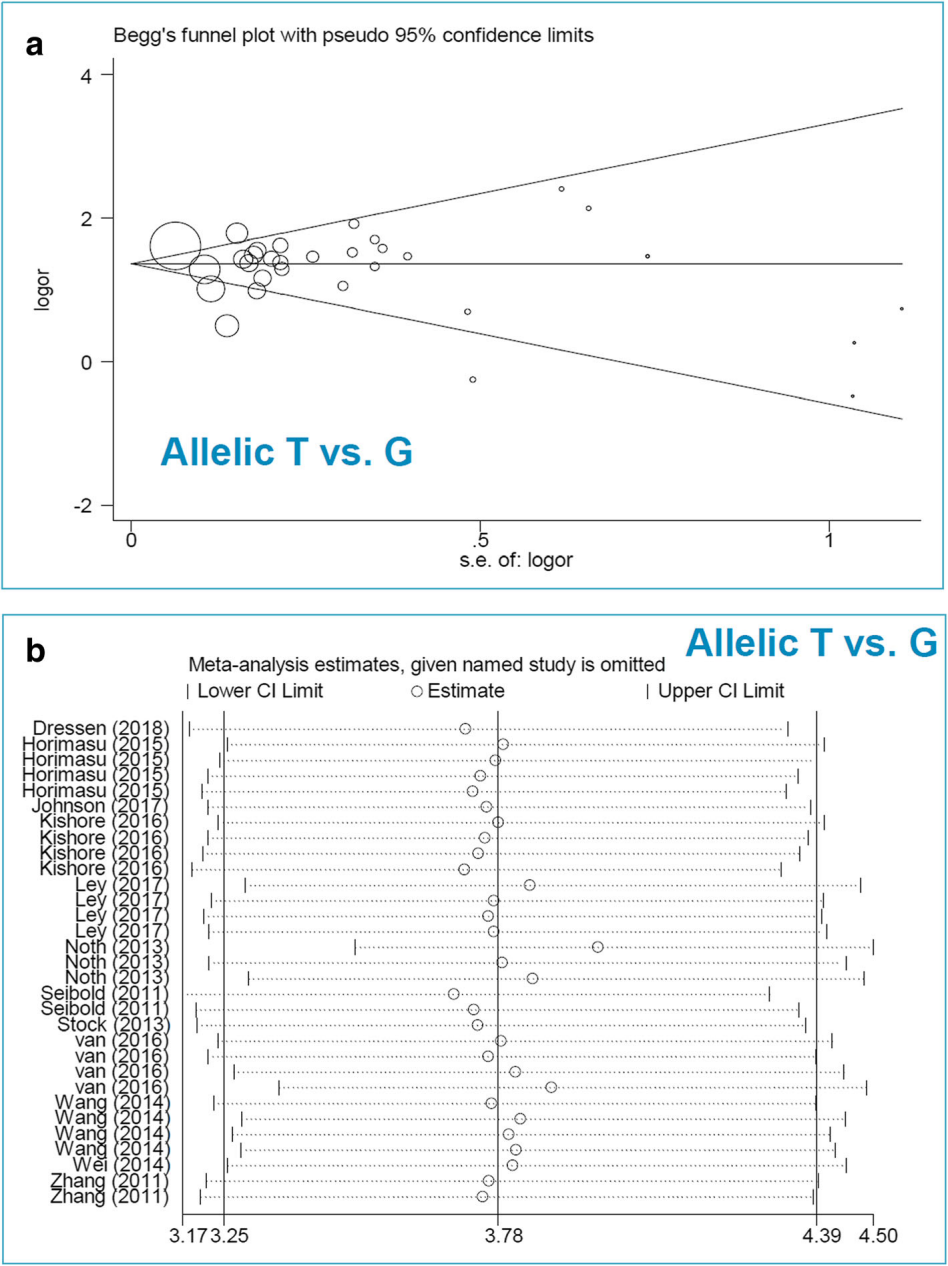
Sensitivity analysis and Begg test plot for *MUC5B* rs35705950 under the allelic model. **a** Sensitivity analysis data; **b** Begg test

As shown in Table 2, we did not observe a large publication bias among the comparisons, as evidenced by *P*_*Begg*_ > 0.05 and *P*_*Egger*_ > 0.05 for all models except the Egger test under GT vs. GG (*P*_*Egger*_ = 0.002) and GT + TT vs. GG (*P*_*Egger*_ = 0.002) for *MUC5B* rs35705950. Fig. 5a and Additional file 2: Figure S7a display the Begg test plot under the allelic model (as an example).

### Sensitivity and TSA data

We observed relatively stable pooling data in the sensitivity analyses of *FCGR2A* rs1801274 (e.g., Additional file 2: Figure S7b for the allelic model). However, our TSA test data (Figure S8) showed that the cumulative Z-curve did not totally reach the conventional boundary and required information size, suggesting the requirement for a larger sample size to confirm the negative association between *FCGR2A* rs1801274 and pneumonia risk.

With regards to *MUC5B* rs35705950, we found that the cumulative Z-curve of GT + TT vs. GG model can cross the TSA monitoring boundary despite not reaching the required information size in the overall population (Additional file 2: Figure S9), Caucasian population (Additional file 2: Figure S10), and Asian population (Additional file 2: Figure S11), indicating the robustness of the conclusions. Furthermore, we also observed the relatively stable or credible outcomes for the *MUC5B* rs35705950 through the sensitivity analyses (e.g., Fig. 5b for the allelic model).

## Discussion

The inconclusive result of the genetic influence of *FCGR2A* rs1801274 on the susceptibility to clinical pneumonia has been observed in previous studies. For example, a negative effect of *FCGR2A* rs1801274 was detected for the development of pneumonia in systemic lupus erythematosus patients [15]; additionally, *FCGR2A* rs1801274 was not found to increase the risk of invasive pneumococcal disease in Belgium [16]. Although *FCGR2A* rs1801274 was not identified in any genome-wide association study (GWAS) of pneumonia [8, 22], we observed a positive association between *FCGR2A* rs1801274 and the risk of pneumonia. *FCGR2A* rs1801274 was reported to be significantly associated with the risk of severe pneumonia in A/H1N1 influenza infection [20], bacteremic pneumococcal pneumonia infection [18], and the severity of community-acquired pneumonia [12]. Here, for the first time, we carried out a meta-analysis and TSA test to comprehensively evaluate the genetic influence of *FCGR2A* rs1801274 in the susceptibility to clinical pneumonia. Upon pooling a total of 1332 cases and 5428 controls from thirteen studies, no strong evidence was found to support a significant association between *FCGR2A* rs1801274 and overall pneumonia risk.

In 2013, Borie, R. et al. included four case-control studies from three articles [23, 24, 44] to perform a meta-analysis of the association between *MUC5B* rs35705950 and idiopathic pulmonary fibrosis risk [24]. A total of nine case-control studies from seven articles [8, 23, 24, 36, 40, 42, 45] were included in another meta-analysis by Zhu, Q. Q. in 2015 [26]; while twelve case-control studies from eight articles [8, 23, 24, 36, 40, 42, 43, 46] were included in an updated meta-analysis by Lee, M. G. et al., 2015 [25]. Given the differences in study selection, basic information extraction, pooling strategies and data description, we carried out a quantitative synthesis to assess the effect of *MUC5B* rs35705950 on the risk of pneumonia diseases, including idiopathic pulmonary fibrosis and nonspecific interstitial pneumonia.

In the present study, we performed the extensive retrieval of three databases (as of 25 February 2020) to capture potential publications for inclusion in a series of pooling analyses. Based on our strict screening strategy, we included a total of 5644 cases and 14,624 controls across thirty-one studies in the overall meta-analysis, followed by subgroup analyses according to the factors of race, control source and pneumonia type. A total of six genetic models, namely, allelic, carrier, homozygotic, heterozygotic, dominant and recessive, were applied. We excluded the studies based on the strict requirements of the HWE principle. Our pooling data suggest that *MUC5B* rs35705950 is closely associated with an increased risk of pneumonia diseases, especially idiopathic pulmonary fibrosis, in both Asians and Caucasians. The positive genetic relationship between *MUC5B* rs35705950 and high susceptibility to idiopathic pulmonary fibrosis is consistent with the results of previous meta-analyses [24–26] and GWAS evidence [8, 22]. When comparing the OR value under different genetic models, the TT genotype carriers were found to be more susceptible to pneumonia disease than the GT genotype carriers. It is possible that the “T” allele of *MUC5B* rs35705950 confers an enhanced susceptibility to pneumonia patients in a dose-dependent manner.

Our study has several advantages over other studies. First, none of the included case-control studies were of low quality based on the assessment analysis of the NOS system. Second, we excluded case-control studies in which the genotypic distribution in the control group did not conform to the HWE principle. Third, we found no obvious evidence of a large publication bias, according to the Begg and Egger analyses. Fourth, we observed the stability of the pooling data when applying our leave-one-out sensitivity analysis, along with the FPRP and TSA analyses. Our study enables readers to understand the current research status of pneumonia-related *MUC5B* rs35705950 in different populations, as well as the pooling evidence based on the currently available data.

However, some disadvantages of our study may affect our statistical power. There was high inter-study heterogeneity in some comparisons. Despite thirteen case-control studies being included in the overall pooling analysis for *FCGR2A* rs1801274, certain subgroups contained small sample sizes. For example, very limited case-control studies in the subgroup analysis of “specific pneumonia type” deterred us from performing the relative analysis. No cases in the homogenous Asian population were enrolled for pooling analysis. Although we observed a negative result in the pooling analysis based on the presently available data, our new TSA test for *FCGR2A* rs1801274 indicated the necessity for a larger sample size to confirm the negative association between *FCGR2A* rs1801274 and pneumonia risk both the Caucasian and Asian populations.

We observed a positive association between *MUC5B* rs35705950 and the overall risk of pneumonia in the Asian population. We also performed FPRP and TSA tests to confirm this relationship. However, there were only five case-control studies, and we could not perform further pooling analysis based on the different phenotypes due to the limited available data. Likewise, only four case-control studies were included in the subgroup analysis of “NSIP”. We failed to detect a genetic influence of *MCU5B* rs35705950 on the risk of other pneumonia types, such as idiopathic interstitial pneumonia, chronic hypersensitivity pneumonitis, and familial interstitial pneumonia.

When more case-control studies become available, it would be meaningful to take additional factors into consideration, such as gender, age, exposure, and drinking/smoking status. As mentioned above, *FCGR2A* rs1801274 and *MUC5B* rs35705950 were selected due to the research status of pneumonia-related variations and the novelty of the data analysis. Thus, there is no obvious correlation between the two. Nevertheless, it is still meaningful to analyze the combined impact of different variants of other genes [e.g., rs2736100 in *TERT* (telomerase reverse transcriptase)] on the susceptibility to the different types of pneumonia in different populations when the data become available.

## Conclusion

Our findings revealed that *MUC5B* rs35705950 is strongly linked to an increased risk of pneumonia diseases in the Asian and Caucasian populations. Owing to data limitations, more available evidence is required to further clarify the genetic relationship between *FCGR2A* rs1801274 and pneumonia susceptibility.

## Supplementary information

**Additional file 1 Table S1**. Detailed terms of database search (as of 25 February 2020). **Table S2**. Allelic and genotypic frequency data of the included case-control studies. **Table S3**. Quality assessment of included case-control studies. **Table S4**. FPRP values for the association between *MUC5B* rs35705950 and pneumonia risk in the Asian population. (DOCX 52.4 KB)

**Additional file 2 Figure S1.** Subgroup analysis data of “control source” for *MUC5B* rs35705950 under the allelic model. **Figure S2.** Subgroup analysis data of “pneumonia type” for *MUC5B* rs35705950 under the carrier model. **Figure S3.** Subgroup analysis data of “pneumonia type” for *MUC5B* rs35705950 under the TT vs. GG model. **Figure S4.** Subgroup analysis data of “pneumonia type” for *MUC5B* rs35705950 under the GT vs. GG model. **Figure S5.** Subgroup analysis data of “pneumonia type” for *MUC5B* rs35705950 under the GT + TT vs. GG model. **Figure S6.** Subgroup analysis data of “pneumonia type” for *MUC5B* rs35705950 under the TT vs. GG + GT model. **Figure S7.** Sensitivity analysis (a) and Begg test data (b) for *FCGR2A* rs1801274 under the allelic T vs. G model. **Figure S8.** TSA data for *FCGR2A* rs1801274 under the AG + GG vs. AA model. **Figure S9.** TSA data for *MUC5B* rs35705950 under the GT + TT vs. GG model in the overall populations; **Figure S10.** TSA data for *MUC5B* rs35705950 under the GT + TT vs. GG model in the Caucasian population; **Figure S11.** TSA data for *MUC5B* rs35705950 under the GT + TT vs. GG model in the Asian population. (PDF 1.9 MB)

## Data Availability

All data generated or analysed during this study are included in this published article and its supplementary information files.

## Abbreviations

FCGR2A: Fc fragment of IgG receptor IIa
MUC5B: Mucin 5B, oligomeric mucus/gel-forming
AIDS: Immune deficiency syndrome
IPF: Idiopathic pulmonary fibrosis
NSIP: Nonspecific interstitial pneumonia
IIP: Idiopathic interstitial pneumonia
PRISMA: Preferred reporting items for systematic reviews and meta-analyses
EMBASE: Excerpta Medica Database
WOS: Web of Science
MeSH: Medical subject headings
HWE: Hardy-Weinberg equilibrium
NOS: Newcastle-Ottawa quality assessment scale
CI: Confidence interval
FDR: False discovery rate
FPRP: False-positive report probability
TSA: Trial sequential analysis
GWAS: Genome-wide association study
TERT: Telomerase reverse transcriptase
