# Supplementary material for: Association between FCGR2A rs1801274 and MUC5B rs35705950 variations and pneumonia susceptibility
Source: BMC Med Genet. 2020 Apr 6;21:71. doi: 10.1186/s12881-020-01005-1 (PMC7137230; doi:10.1186/s12881-020-01005-1)
Supplement: Supplementary file 1 — Additional file 1 Table S1. Detailed terms of database search (as of 25 February 2020). Table S2. Allelic and genotypic frequency data of the included case-control studies. Table S3. Quality assessment of included case-control studies. Table S4. FPRP values for the association between MUC5B rs35705950 and pneumonia risk in the Asian population. (DOCX 52.4 KB) [file 12881_2020_1005_MOESM1_ESM.docx]

### Table S1 Detailed terms of database search (as of 25 February 2020)

| **Database** | **Retrieval steps** | **Searching Terms** | **Number** |
| --- | --- | --- | --- |
| **Pubmed** | **#1** | ((("pneumonia"[MeSH Terms] OR "pneumonia"[All Fields]) OR ("lobar"[All Fields] AND "pneumonia"[All Fields])) OR "lobar pneumonia"[All Fields])) OR ((("pneumonia"[MeSH Terms] OR "pneumonia"[All Fields]) OR ("lobar"[All Fields] AND "pneumonias"[All Fields])) OR "lobar pneumonias"[All Fields])) OR ((("pneumonia"[MeSH Terms] OR "pneumonia"[All Fields]) OR ("pneumonias"[All Fields] AND "lobar"[All Fields])) )) OR ((("pneumonia"[MeSH Terms] OR "pneumonia"[All Fields]) OR ("pneumonia"[All Fields] AND "lobar"[All Fields])) OR "pneumonia lobar"[All Fields])) OR ((("pneumonia"[MeSH Terms] OR "pneumonia"[All Fields]) OR (("experimental"[All Fields] AND "lung"[All Fields]) AND "inflammation"[All Fields])) OR "experimental lung inflammation"[All Fields])) OR ((("pneumonia"[MeSH Terms] OR "pneumonia"[All Fields]) OR (("experimental"[All Fields] AND "lung"[All Fields]) AND "inflammations"[All Fields])) )) OR ((("pneumonia"[MeSH Terms] OR "pneumonia"[All Fields]) OR (("inflammation"[All Fields] AND "experimental"[All Fields]) AND "lung"[All Fields])) )) OR ((("pneumonia"[MeSH Terms] OR "pneumonia"[All Fields]) OR (("lung"[All Fields] AND "inflammation"[All Fields]) AND "experimental"[All Fields])) OR "lung inflammation experimental"[All Fields])) OR ((("pneumonia"[MeSH Terms] OR "pneumonia"[All Fields]) OR (("lung"[All Fields] AND "inflammations"[All Fields]) AND "experimental"[All Fields])) )) OR (("pneumonia"[MeSH Terms] OR "pneumonia"[All Fields]) OR "pneumonitis"[All Fields])) OR (("pneumonia"[MeSH Terms] OR "pneumonia"[All Fields]) OR "pneumonitides"[All Fields])) OR ((("pneumonia"[MeSH Terms] OR "pneumonia"[All Fields]) OR ("pulmonary"[All Fields] AND "inflammation"[All Fields])) OR "pulmonary inflammation"[All Fields])) OR ((("pneumonia"[MeSH Terms] OR "pneumonia"[All Fields]) OR ("inflammation"[All Fields] AND "pulmonary"[All Fields])) OR "inflammation pulmonary"[All Fields])) OR ((("pneumonia"[MeSH Terms] OR "pneumonia"[All Fields]) OR ("inflammations"[All Fields] AND "pulmonary"[All Fields])) OR "inflammations pulmonary"[All Fields])) OR ((("pneumonia"[MeSH Terms] OR "pneumonia"[All Fields]) OR ("pulmonary"[All Fields] AND "inflammations"[All Fields])) OR "pulmonary inflammations"[All Fields])) OR ((("pneumonia"[MeSH Terms] OR "pneumonia"[All Fields]) OR ("lung"[All Fields] AND "inflammation"[All Fields])) OR "lung inflammation"[All Fields])) OR ((("pneumonia"[MeSH Terms] OR "pneumonia"[All Fields]) OR ("inflammation"[All Fields] AND "lung"[All Fields])) OR "inflammation lung"[All Fields])) OR ((("pneumonia"[MeSH Terms] OR "pneumonia"[All Fields]) OR ("inflammations"[All Fields] AND "lung"[All Fields])) OR "inflammations lung"[All Fields])) OR ((("pneumonia"[MeSH Terms] OR "pneumonia"[All Fields]) OR ("lung"[All Fields] AND "inflammations"[All Fields])) OR "lung inflammations"[All Fields]) | [**273,197**](https://www.ncbi.nlm.nih.gov/pubmed/?cmd=HistorySearch&querykey=10) |
|  | **#2** | ((((((((((((((((((("FCGR2A"[All Fields] OR ((((("immunoglobulin fc fragments"[MeSH Terms] OR (("immunoglobulin"[All Fields] AND "Fc"[All Fields]) AND "fragments"[All Fields])) OR "immunoglobulin fc fragments"[All Fields]) OR ("Fc"[All Fields] AND "fragment"[All Fields])) OR "fc fragment"[All Fields]) AND "IgG"[All Fields] AND (("receptor"[All Fields] OR "receptor s"[All Fields]) OR "receptors"[All Fields]) AND "IIa"[All Fields])) OR ("Low"[All Fields] AND (("affinant"[All Fields] OR "affinities"[All Fields]) OR "affinity"[All Fields]) AND ((((("immunoglobulin s"[All Fields] OR "immunoglobuline"[All Fields]) OR "immunoglobulines"[All Fields]) OR "immunoglobulins"[MeSH Terms]) OR "immunoglobulins"[All Fields]) OR "immunoglobulin"[All Fields]) AND (((((("gamma rays"[MeSH Terms] OR ("gamma"[All Fields] AND "rays"[All Fields])) OR "gamma rays"[All Fields]) OR "gamma"[All Fields]) OR "gamma s"[All Fields]) OR "gammae"[All Fields]) OR "gammas"[All Fields]) AND "Fc"[All Fields] AND ((((((((((((("geographic locations"[MeSH Terms] OR ("geographic"[All Fields] AND "locations"[All Fields])) OR "geographic locations"[All Fields]) OR "region"[All Fields]) OR "region s"[All Fields]) OR "regional"[All Fields]) OR "regionalization"[All Fields]) OR "regionalizations"[All Fields]) OR "regionalize"[All Fields]) OR "regionalized"[All Fields]) OR "regionalizing"[All Fields]) OR "regionally"[All Fields]) OR "regionals"[All Fields]) OR "regions"[All Fields]) AND (("receptor"[All Fields] OR "receptor s"[All Fields]) OR "receptors"[All Fields]) AND "II-A"[All Fields])) OR ((((("immunoglobulin fc fragments"[MeSH Terms] OR (("immunoglobulin"[All Fields] AND "Fc"[All Fields]) AND "fragments"[All Fields])) OR "immunoglobulin fc fragments"[All Fields]) OR ("Fc"[All Fields] AND "fragment"[All Fields])) OR "fc fragment"[All Fields]) AND "IgG"[All Fields] AND "Low"[All Fields] AND (("affinant"[All Fields] OR "affinities"[All Fields]) OR "affinity"[All Fields]) AND "IIa"[All Fields] AND (("receptor"[All Fields] OR "receptor s"[All Fields]) OR "receptors"[All Fields]))) AND "CD32"[All Fields]) OR (("immunoglobulin g"[MeSH Terms] OR "immunoglobulin g"[All Fields]) AND (((("receptors, igg"[MeSH Terms] OR ("receptors"[All Fields] AND "IgG"[All Fields])) OR "igg receptors"[All Fields]) OR (("Fc"[All Fields] AND "receptor"[All Fields]) AND "ii"[All Fields])) OR "fc receptor ii"[All Fields]))) OR ("IgG"[All Fields] AND (((("receptors, fc"[MeSH Terms] OR ("receptors"[All Fields] AND "Fc"[All Fields])) OR "fc receptors"[All Fields]) OR ("Fc"[All Fields] AND "receptor"[All Fields])) OR "fc receptor"[All Fields]) AND "II-A"[All Fields])) OR (("fc gamma receptor iia"[Supplementary Concept] OR "fc gamma receptor iia"[All Fields]) OR "fc gamma riia"[All Fields])) OR "CDw32"[All Fields]) OR "IGFR2"[All Fields]) OR "FCG2"[All Fields]) OR "CD32"[All Fields]) OR ((((("immunoglobulin fc fragments"[MeSH Terms] OR (("immunoglobulin"[All Fields] AND "Fc"[All Fields]) AND "fragments"[All Fields])) OR "immunoglobulin fc fragments"[All Fields]) OR ("Fc"[All Fields] AND "fragment"[All Fields])) OR "fc fragment"[All Fields]) AND "IgG"[All Fields] AND "Low"[All Fields] AND (("affinant"[All Fields] OR "affinities"[All Fields]) OR "affinity"[All Fields]) AND "IIa"[All Fields] AND (("receptor"[All Fields] OR "receptor s"[All Fields]) OR "receptors"[All Fields]))) AND "CD32"[All Fields]) OR (((("receptors, igg"[MeSH Terms] OR ("receptors"[All Fields] AND "IgG"[All Fields])) OR "igg receptors"[All Fields]) OR (("Fc"[All Fields] AND "gamma"[All Fields]) AND "receptor"[All Fields])) OR "fc gamma receptor"[All Fields])) OR (("fc gamma receptor iia"[Supplementary Concept] OR "fc gamma receptor iia"[All Fields]) OR "fc gamma receptor iia"[All Fields])) OR ("Fc-Gamma"[All Fields] AND "RII-A"[All Fields])) OR ("CD32"[All Fields] AND (((((((((("antigen s"[All Fields] OR "antigene"[All Fields]) OR "antigenes"[All Fields]) OR "antigenic"[All Fields]) OR "antigenically"[All Fields]) OR "antigenicities"[All Fields]) OR "antigenicity"[All Fields]) OR "antigenized"[All Fields]) OR "antigens"[MeSH Terms]) OR "antigens"[All Fields]) OR "antigen"[All Fields]))) OR "CD32A"[All Fields]) OR "FCGR2"[All Fields]) OR ((("receptors, igg"[MeSH Terms] OR ("receptors"[All Fields] AND "IgG"[All Fields])) OR "igg receptors"[All Fields]) OR "fcgr"[All Fields]) | **23,056** |
|  | **#3** | ((((((((((((("mucin-5b"[MeSH Terms] OR "mucin 5b"[All Fields]) OR "muc5b"[All Fields]) OR (((("mucin-5b"[MeSH Terms] OR "mucin 5b"[All Fields]) OR ("mucin"[All Fields] AND "5b"[All Fields])) OR "mucin 5b"[All Fields]) AND (((((("oligomeric"[All Fields] OR "oligomerization"[All Fields]) OR "oligomerizations"[All Fields]) OR "oligomerize"[All Fields]) OR "oligomerized"[All Fields]) OR "oligomerizes"[All Fields]) OR "oligomerizing"[All Fields]) AND "mucus gel forming"[All Fields])) OR ((("mucin-5b"[MeSH Terms] OR "mucin 5b"[All Fields]) OR ((((("high"[All Fields] AND "molecular"[All Fields]) AND "weight"[All Fields]) AND "salivary"[All Fields]) AND "mucin"[All Fields]) AND "MG1"[All Fields])) OR "high molecular weight salivary mucin mg1"[All Fields])) OR (((((("mucine"[All Fields] OR "mucines"[All Fields]) OR "mucinous"[All Fields]) OR "mucins"[MeSH Terms]) OR "mucins"[All Fields]) OR "mucin"[All Fields]) AND "5"[All Fields] AND (((("subtype"[All Fields] OR "subtyped"[All Fields]) OR "subtypes"[All Fields]) OR "subtyping"[All Fields]) OR "subtypings"[All Fields]) AND "B"[All Fields] AND "Tracheobronchial"[All Fields])) OR ((("mucin-5b"[MeSH Terms] OR "mucin 5b"[All Fields]) OR (("sublingual"[All Fields] AND "gland"[All Fields]) AND "mucin"[All Fields])) )) OR ((("mucin-5b"[MeSH Terms] OR "mucin 5b"[All Fields]) OR ("mucin"[All Fields] AND "5b"[All Fields])) OR "mucin 5b"[All Fields])) OR ((("mucin-5b"[MeSH Terms] OR "mucin 5b"[All Fields]) OR ("muc"[All Fields] AND "5b"[All Fields])) OR "muc 5b"[All Fields])) OR "MUC5"[All Fields]) OR ("Mucin-5"[All Fields] AND (((("subtype"[All Fields] OR "subtyped"[All Fields]) OR "subtypes"[All Fields]) OR "subtyping"[All Fields]) OR "subtypings"[All Fields]) AND "B"[All Fields] AND "Tracheobronchial"[All Fields])) OR ((("mucin-5b"[MeSH Terms] OR "mucin 5b"[All Fields]) OR (("cervical"[All Fields] AND "mucin"[All Fields]) AND "muc5b"[All Fields])) )) OR ((("mucin-5b"[MeSH Terms] OR "mucin 5b"[All Fields]) OR ("cervical"[All Fields] AND "mucin"[All Fields])) OR "cervical mucin"[All Fields])) OR "MUC9"[All Fields]) OR "MG1"[All Fields] | **9,861** |
|  | **#4** | #2 OR #3 | **31,915** |
|  | **#5** | #2 OR #3 | **610** |
|  | **#6** | ((((((((((((((("polymorphism, genetic"[MeSH Terms] OR ("polymorphism"[All Fields] AND "genetic"[All Fields])) OR "genetic polymorphism"[All Fields]) OR ("polymorphism"[All Fields] AND "genetic"[All Fields])) OR "polymorphism genetic"[All Fields]) OR (((("polymorphism, genetic"[MeSH Terms] OR ("polymorphism"[All Fields] AND "genetic"[All Fields])) OR "genetic polymorphism"[All Fields]) OR ("polymorphisms"[All Fields] AND "genetic"[All Fields])) OR "polymorphisms genetic"[All Fields])) OR (((("polymorphism, genetic"[MeSH Terms] OR ("polymorphism"[All Fields] AND "genetic"[All Fields])) OR "genetic polymorphism"[All Fields]) OR ("genetic"[All Fields] AND "polymorphisms"[All Fields])) OR "genetic polymorphisms"[All Fields])) OR ((("polymorphism, genetic"[MeSH Terms] OR ("polymorphism"[All Fields] AND "genetic"[All Fields])) OR "genetic polymorphism"[All Fields]) OR ("genetic"[All Fields] AND "polymorphism"[All Fields]))) OR ((((((("polymorphic"[All Fields] OR "polymorphics"[All Fields]) OR "polymorphism s"[All Fields]) OR "polymorphism, genetic"[MeSH Terms]) OR ("polymorphism"[All Fields] AND "genetic"[All Fields])) OR "genetic polymorphism"[All Fields]) OR "polymorphism"[All Fields]) OR "polymorphisms"[All Fields])) AND (((((((((((("genetic therapy"[MeSH Terms] OR ("genetic"[All Fields] AND "therapy"[All Fields])) OR "genetic therapy"[All Fields]) OR "genetic"[All Fields]) OR "genetical"[All Fields]) OR "genetically"[All Fields]) OR "genetics"[MeSH Subheading]) OR "genetics"[All Fields]) OR "genetics"[MeSH Terms]) OR "viverridae"[MeSH Terms]) OR "viverridae"[All Fields]) OR "genet"[All Fields]) OR "genets"[All Fields])) OR ((((((("polymorphic"[All Fields] OR "polymorphics"[All Fields]) OR "polymorphism s"[All Fields]) OR "polymorphism, genetic"[MeSH Terms]) OR ("polymorphism"[All Fields] AND "genetic"[All Fields])) OR "genetic polymorphism"[All Fields]) OR "polymorphism"[All Fields]) OR "polymorphisms"[All Fields])) AND (((((((((((("genetic therapy"[MeSH Terms] OR ("genetic"[All Fields] AND "therapy"[All Fields])) OR "genetic therapy"[All Fields]) OR "genetic"[All Fields]) OR "genetical"[All Fields]) OR "genetically"[All Fields]) OR "genetics"[MeSH Subheading]) OR "genetics"[All Fields]) OR "genetics"[MeSH Terms]) OR "viverridae"[MeSH Terms]) OR "viverridae"[All Fields]) OR "genet"[All Fields]) OR "genets"[All Fields])) OR ("socioaffect neurosci psychol"[Journal] OR "snp"[All Fields])) OR (((((((((("mutate"[All Fields] OR "mutated"[All Fields]) OR "mutates"[All Fields]) OR "mutating"[All Fields]) OR "mutation"[MeSH Terms]) OR "mutation"[All Fields]) OR "mutations"[All Fields]) OR "mutation s"[All Fields]) OR "mutational"[All Fields]) OR "mutator"[All Fields]) OR "mutators"[All Fields])) OR (((((((((("mutate"[All Fields] OR "mutated"[All Fields]) OR "mutates"[All Fields]) OR "mutating"[All Fields]) OR "mutation"[MeSH Terms]) OR "mutation"[All Fields]) OR "mutations"[All Fields]) OR "mutation s"[All Fields]) OR "mutational"[All Fields]) OR "mutator"[All Fields]) OR "mutators"[All Fields])) OR ((((((("polymorphic"[All Fields] OR "polymorphics"[All Fields]) OR "polymorphism s"[All Fields]) OR "polymorphism, genetic"[MeSH Terms]) OR ("polymorphism"[All Fields] AND "genetic"[All Fields])) OR "genetic polymorphism"[All Fields]) OR "polymorphism"[All Fields]) OR "polymorphisms"[All Fields])) OR ((((((("polymorphic"[All Fields] OR "polymorphics"[All Fields]) OR "polymorphism s"[All Fields]) OR "polymorphism, genetic"[MeSH Terms]) OR ("polymorphism"[All Fields] AND "genetic"[All Fields])) OR "genetic polymorphism"[All Fields]) OR "polymorphism"[All Fields]) OR "polymorphisms"[All Fields]) | **1,386,751** |
|  | **#7** | #5 and #6 | **87** |
| **EMBASE** | **#1** | 'mucb2' OR 'fcgr2a' | **524** |
|  | **#2** | 'genetic polymorphism'/exp OR 'genetic polymorphism' OR 'polymorphism (genetics)'/exp OR 'polymorphism (genetics)' OR 'polymorphism, genetic'/exp OR 'polymorphism, genetic' OR 'polymorphism'/exp OR 'polymorphism' OR 'polymorphisms' | **523,184** |
|  | **#3** | 'pneumonia'/exp OR 'pneumonia' OR 'acute diffuse pneumonia'/exp OR 'acute diffuse pneumonia' OR 'atrophic reticular pneumonia'/exp OR 'atrophic reticular pneumonia' OR 'enzootic pneumonia'/exp OR 'enzootic pneumonia' OR 'inflammation, lung'/exp OR 'inflammation, lung' OR 'inflammatory lung disease'/exp OR 'inflammatory lung disease' OR 'lobitis'/exp OR 'lobitis' OR 'lung inflammation'/exp OR 'lung inflammation' OR 'nonspecific inflammatory lung disease'/exp OR 'nonspecific inflammatory lung disease' OR 'peripneumonia'/exp OR 'peripneumonia' OR 'pleurisy, pneumonia'/exp OR 'pleurisy, pneumonia' OR 'pleuritic pneumonia'/exp OR 'pleuritic pneumonia' OR 'pleuritis, pneumonia'/exp OR 'pleuritis, pneumonia' OR 'pleuropneumonia'/exp OR 'pleuropneumonia' OR 'pleuropneumonitis'/exp OR 'pleuropneumonitis' OR 'pneumonia pleuritica'/exp OR 'pneumonia pleuritica' OR 'pneumonia superficialis'/exp OR 'pneumonia superficialis' OR 'pneumonic lung'/exp OR 'pneumonic lung' OR 'pneumonic pleurisy'/exp OR 'pneumonic pleurisy' OR 'pneumonic pleuritis'/exp OR 'pneumonic pleuritis' OR 'pneumonitis'/exp OR 'pneumonitis' OR 'pulmonal inflammation'/exp OR 'pulmonal inflammation' OR 'pulmonary inflammation'/exp OR 'pulmonary inflammation' OR 'pulmonic inflammation'/exp OR 'pulmonic inflammation' OR 'stable pneumonia'/exp OR 'stable pneumonia' OR 'superficial pneumonia'/exp OR 'superficial pneumonia' | **353,704** |
|  | **#4** | #1 AND #2 AND #3 | **18** |
| **WOS** | **#1** | TOPIC: (Pneumonia) OR TOPIC: (Pneumonias) OR TOPIC: (Pneumonitis) OR TOPIC: (Pneumonitides) OR TOPIC: (Lung Inflammations)  Databases= WOS, KJD, MEDLINE, RSCI, SCIELO Timespan=All years  Search language=Auto | **327,016** |
|  | **#2** | TOPIC: (FCGR2A) OR TOPIC: (Fc Fragment of IgG Receptor IIa) OR TOPIC: (MUC5B) OR TOPIC: (Mucin-5B)  Databases= WOS, KJD, MEDLINE, RSCI, SCIELO Timespan=All years  Search language=Auto | **2,144** |
|  | **#3** | TOPIC: (Genetic Polymorphisms) OR TOPIC: (Genetic Polymorphism) OR TOPIC: (SNP) OR TOPIC: (Mutation) OR TOPIC: (Mutations) OR TOPIC: (Polymorphism) OR TOPIC: (Polymorphism) OR TOPIC: (variant) OR TOPIC: (variants)  Databases= WOS, KJD, MEDLINE, RSCI, SCIELO Timespan=All years  Search language=Auto | **2,215,759** |
|  | **#4** | #1 AND #2 AND #3 | **158** |

*EMBASE* Excerpta Medica Database, *WOS* Web of Science.

### Table S2 Allele and genotypic frequency data of the included case-control studies

| **First author, Year [Ref.]** | **Variation** | **Case** | | | **Disease type** | **Control** | | |
| --- | --- | --- | --- | --- | --- | --- | --- | --- |
|  |  | **MM/Mm/mm** | **M/m** | **Total** |  | **MM/Mm/mm** | **M/m** | **Total** |
| Bougle, 2012 [11] | rs1801274 | 155^&^/43 | NA | 198 | ICU /pneummonia | 2092^&^/697 | 2817/2761 | 2789 |
| Dressen, 2018 [35] | rs35705950 | 464/910/136 | 1838/1182 | 1510 | IPF | 1472/376/26 | 3320/428 | 1874 |
| Endeman, 2009 [12] | rs1801274 | 56/91/53 | 203/197 | 200 | CAP | 75/161/77 | 311/315 | 313 |
| Forthal, 2007 [13] | rs1801274 | 31/35/14 | 97/63 | 80 | AIDS/ pneumonia | 118/227/133 | 463/493 | 478 |
| Horimasu, 2015 [36] | rs35705950 | 29/1/0 | 59/1 | 30 | NSIP | 305/5/0 | 615/5 | 310 |
|  |  | 41/3/0 | 85/3 | 44 | IPF | 305/5/0 | 615/5 | 310 |
|  | rs35705950 | 16/13/2 | 45/17 | 31 | NSIP | 32/3/0 | 67/3 | 35 |
|  |  | 32/31/8 | 95/47 | 71 | IPF | 32/3/0 | 67/3 | 35 |
| Johnson, 2017 [37] | rs35705950 | NA | 89/31 | 60 | IIP | NA | 249/19 | 134 |
| Jonsson, 2006 [14] | rs1801274 | 7/17/6 | 31/29 | 30 | C2D/pneumonia | 45/103/52 | 193/207 | 200 |
| Kinder, 2007 [15] | rs1801274 | 14/7/21 | 35/49 | 42 | SLE/pneumonia | 46/96/75 | 188/246 | 217 |
| Kishore, 2016 [38] | rs35705950 | 21/17/3 | 59/23 | 41 | IPF^a^ | 80/14/2 | 174/18 | 96 |
|  | rs35705950 | 12/20/1 | 44/22 | 33 | IPF^b^ | 80/14/2 | 174/18 | 96 |
|  | rs35705950 | 16/14/6 | 46/26 | 36 | IPF^c^ | 80/14/2 | 174/18 | 96 |
|  | rs35705950 | 18/24/9 | 60/42 | 51 | IPF^d^ | 80/14/2 | 174/18 | 96 |
| Ley, 2017 [39] | rs35705950 | NA | 186/60 | 123 | CHP^e^ | NA | 898/108 | 503 |
|  |  | NA | 88/42 | 65 | CHP^f^ | NA | 898/108 | 503 |
|  |  | NA | 196/98 | 147 | IPF^e^ | NA | 898/108 | 503 |
|  |  | NA | 171/81 | 126 | IPF^f^ | NA | 898/108 | 503 |
| Moens, 2006 [16] | rs1801274 | 14/29/12 | 57/43 | 55 | pneumonia | 22/47/31 | 91/109 | 100 |
| Noth, 2013 [8] | rs35705950 | NA | 932/152 | 542 | IPF^g^ | NA | 986/98 | 542 |
|  |  | NA | 729/359 | 544 | IPF^h^ | NA | 1209/165 | 687 |
|  |  | NA | 447/201 | 324 | IPF^i^ | NA | 1207/197 | 702 |
| Seibold, 2011 [23] | rs35705950 | NA | 615/369 | 492 | IPF | NA | 585/59 | 322 |
|  |  | NA | 110/56 | 83 | FIP | NA | 585/59 | 322 |
| Sole, 2011 [17] | rs1801274 | 67/166/86 | 300/338 | 319 | pneumococcal CAP | 284/630/310 | 1198/1250 | 1224 |
|  |  | 37/129/68 | 203/265 | 234 | nonbacteremic CAP | 284/630/310 | 1198/1250 | 1224 |
|  |  | 30/37/18 | 97/73 | 85 | bacteremic CAP | 284/630/310 | 1198/1250 | 1224 |
| Stock, 2013 [40] | rs35705950 | 42/58/10 | 142/78 | 110 | IPF | 337/71/8 | 745/87 | 416 |
| van, 2016 [41] | rs35705950 | 59/51/5 | 169/61 | 115 | spIPF | 205/43/1 | 453/45 | 249 |
|  |  | 24/29/2 | 77/33 | 55 | FIP | 205/43/1 | 453/45 | 249 |
|  |  | 26/15/2 | 67/19 | 43 | iNSIP | 205/43/1 | 453/45 | 249 |
|  |  | 30/5/0 | 65/5 | 35 | CTD-IP | 205/43/1 | 453/45 | 249 |
| Wang, 2014 [42] | rs35705950 | 154/11/0 | 319/11 | 165 | IPF | 997/16/0 | 2010/16 | 1013 |
|  |  | 185/6/0 | 376/6 | 191 | CTD-NSIP | 997/16/0 | 2010/16 | 1013 |
|  |  | 48/1/0 | 97/1 | 49 | CTD-UIP | 997/16/0 | 2010/16 | 1013 |
|  |  | 102/1/0 | 205/1 | 103 | pneumonia | 997/16/0 | 2010/16 | 1013 |
| Wei, 2014 [43] | rs35705950 | 37/44/3 | 118/50 | 84 | IPF | 539/139/11 | 1217/161 | 689 |
| Yee, 2000 [18] | rs1801274 | 7/14/21 | 28/56 | 42 | bacteremic pneumonia | 34/62/40 | 130/142 | 136 |
|  |  | 10/10/8 | 30/26 | 28 | nonbacteremic pneumonia | 34/62/40 | 130/142 | 136 |
| Yuan, 2003 [19] | rs1801274 | 14/22/27 | 50/76 | 63 | pneumonia | 4/10/6 | 18/22 | 20 |
|  |  | 14/22/27 | 50/76 | 63 | pneumonia | 16/30/12 | 62/54 | 58 |
| Zhang, 2011 [44] | rs35705950 | 95/136/15 | 326/166 | 246 | IPF^j^ | 132/32/2 | 296/36 | 166 |
|  |  | 36/50/9 | 122/68 | 95 | IPF^k^ | 504/122/10 | 1130/142 | 636 |
| Zuniga, 2012 [20] | rs1801274 | 12/46/33 | 70/112 | 91 | pneumonia | 34/51/13 | 119/77 | 98 |

*Ref.* reference, *M* major allele (A for rs1801274; G for rs35705950), *m* minor allele (G for rs1801274; T for rs35705950), *ICU* intensive care unit, *IPF* idiopathic pulmonary fibrosis, *CAP* community-acquired pneumonia, *AIDS* acquired immune deficiency syndrome, *NSIP* nonspecific interstitial pneumonia, *IIP* idiopathic interstitial pneumonia, *C2D*, homozygous C2 deficiency, *SLE* systemic lupus erythematosus, *CHP* chronic hypersensitivity pneumonitis, *FIP* familial interstitial pneumonia, *spIPF* sporadic type of idiopathic pulmonary fibrosis, *iNSIP* idiopathic non-specific interstitial pneumonia, *CTD-IP* connective tissue disease associated intersititial pneumonia, *CTD-NSIP* connective tissue diseases-nonspecific interstitial pneumonia pattern, *CTD-UIP* connective tissue diseases-usual interstitial pneumonia pattern, NA not available.

*a* data of Czech Republic, *b* data of Germany, *c* data of Greece, *d* data of France,

*e* data of University of California San Francisco, *f* data of University of Texas Southwestern,

*g* stage one of genome-wide association study, *h* stage two of genome-wide association study,

*i* stage three of genome-wide association study, *j* data of University of Pittsburgh, k, data of University of Chicago,

*&* combined genotype frequency data of MM+Mm.

### Table S3 Quality assessment of included case-control studies

| **First author** | **Year** | **T1** | **T2** | **T3** | **T4** | **T5** | **T6** | **T7** | **T8** | **T9** | **NOS score** |
| --- | --- | --- | --- | --- | --- | --- | --- | --- | --- | --- | --- |
| **Bougle** | **2012** | **➊** | **➊** | **🄌** | **🄌** | **🄌** | **➊** | **➊** | **➊** | **➊** | **6** |
| **Dressen** | **2018** | **➊** | **➊** | **🄌** | **🄌** | **➊** | **➊** | **➊** | **➊** | **➊** | **7** |
| **Endeman** | **2009** | **➊** | **➊** | **➊** | **➊** | **🄌** | **➊** | **🄌** | **➊** | **➊** | **7** |
| **Forthal** | **2007** | **➊** | **➊** | **🄌** | **🄌** | **🄌** | **➊** | **➊** | **➊** | **➊** | **6** |
| **Horimasu** | **2015** | **➊** | **➊** | **➊** | **➊** | **🄌** | **➊** | **➊** | **➊** | **➊** | **8** |
| **Johnson** | **2017** | **➊** | **➊** | **🄌** | **🄌** | **🄌** | **➊** | **🄌** | **➊** | **➊** | **5** |
| **Jonsson** | **2006** | **➊** | **➊** | **➊** | **➊** | **🄌** | **➊** | **🄌** | **➊** | **➊** | **7** |
| **Kinder** | **2007** | **➊** | **➊** | **🄌** | **🄌** | **🄌** | **➊** | **➊** | **➊** | **🄌** | **5** |
| **Kishore** | **2016** | **➊** | **➊** | **➊** | **➊** | **🄌** | **🄌** | **➊** | **➊** | **➊** | **7** |
| **Ley** | **2017** | **➊** | **➊** | **➊** | **➊** | **🄌** | **🄌** | **➊** | **➊** | **🄌** | **6** |
| **Moens** | **2006** | **➊** | **➊** | **🄌** | **🄌** | **➊** | **➊** | **🄌** | **➊** | **➊** | **6** |
| **Noth** | **2013** | **➊** | **➊** | **➊** | **➊** | **➊** | **➊** | **➊** | **➊** | **🄌** | **8** |
| **Seibold** | **2011** | **➊** | **➊** | **➊** | **➊** | **🄌** | **🄌** | **➊** | **➊** | **➊** | **7** |
| **Sole** | **2011** | **➊** | **➊** | **🄌** | **➊** | **🄌** | **➊** | **🄌** | **➊** | **➊** | **6** |
| **Stock** | **2013** | **➊** | **➊** | **➊** | **➊** | **➊** | **🄌** | **🄌** | **➊** | **➊** | **7** |
| **van** | **2016** | **➊** | **➊** | **➊** | **➊** | **🄌** | **➊** | **➊** | **➊** | **➊** | **8** |
| **Wang** | **2014** | **➊** | **➊** | **➊** | **➊** | **➊** | **➊** | **🄌** | **➊** | **➊** | **8** |
| **Wei** | **2014** | **➊** | **➊** | **➊** | **➊** | **➊** | **🄌** | **🄌** | **➊** | **➊** | **7** |
| **Yee** | **2000** | **➊** | **➊** | **🄌** | **🄌** | **🄌** | **➊** | **➊** | **➊** | **➊** | **6** |
| **Yuan** | **2003** | **➊** | **➊** | **➊** | **🄌** | **🄌** | **➊** | **🄌** | **➊** | **➊** | **6** |
| **Zhang** | **2011** | **➊** | **➊** | **➊** | **➊** | **🄌** | **➊** | **🄌** | **➊** | **➊** | **7** |
| **Zuniga** | **2012** | **➊** | **➊** | **➊** | **➊** | **🄌** | **➊** | **➊** | **➊** | **➊** | **8** |

*T1* (Term 1) Is ths case definition adequate?

*T2* (Term 2) Representativeness of cases?

*T3* (Term 3) Selection of controls-community controls?

*T4* (Term 4) Definition of controls-no history of disease?

*T5* (Term 5) Comparability of cases and controls (most important factor)?

*T6* (Term 6) Comparability of cases and controls (any additional factor)?

*T7* (Term 7) secure record of exposure-secure record?

*T8* (Term 8) same method of ascertainment for cases and controls?

*T9* (Term 9) Non-response rate-same rate for both groups?

**➊** 1 score, **🄌**: 0 score, *NOS* Newcastle-Ottawa quality assessment Scale.

### Table S4 FPRP values for the association between *MUC5B* rs35705950 and pneumonia risk in the Asian population

| **Model** | **OR (95% CI)** | ***P^@^*** | **Prior probability level** | | | | | |
| --- | --- | --- | --- | --- | --- | --- | --- | --- |
|  |  |  | **0.25** | **0.1** | **0.01** | **0.001** | **0.0001** | **0.00001** |
| **Allelic T vs. G** | 2.76 [1.67,4.56] | <0.0001 | **0.0250** | **0.0715** | 0.4585 | 0.8952 | 0.9884 | 0.9988 |
| **Carrier T vs. G** | 2.47 [1.52,4.02] | 0.0003 | **0.0354** | **0.0993** | 0.5481 | 0.9245 | 0.9919 | 0.9992 |
| **GT vs. GG** | 2.78 [1.66,4.65] | <0.0001 | **0.0304** | **0.0860** | 0.5481 | 0.9126 | 0.9905 | 0.9990 |
| **GT+TT vs. GG** | 2.78 [1.66,4.65] | <0.0001 | **0.0304** | **0.0860** | 0.5481 | 0.9126 | 0.9905 | 0.9990 |

*OR* odds ratio, *CI* 95% confidence interval, *P****^@^*** *P* value in Chi-square test for genotype frequency distributions,

FPRP value < 0.2 in bold.
