# Supplementary material for: Association between FCGR2A rs1801274 and MUC5B rs35705950 variations and pneumonia susceptibility
Source: BMC Med Genet. 2020 Apr 6;21:71. doi: 10.1186/s12881-020-01005-1 (PMC7137230; doi:10.1186/s12881-020-01005-1)
Supplement: Supplementary file 2 — Additional file 2 Figure S1. Subgroup analysis data of “control source” for MUC5B rs35705950 under the allelic model. Figure S2. Subgroup analysis data of “pneumonia type” for MUC5B rs35705950 under the carrier model. Figure S3. Subgroup analysis data of “pneumonia type” for MUC5B rs35705950 under the TT vs. GG model. Figure S4. Subgroup analysis data of “pneumonia type” for MUC5B rs35705950 under the GT vs. GG model. Figure S5. Subgroup analysis data of “pneumonia type” for MUC5B rs35705950 under the GT + TT vs. GG model. Figure S6. Subgroup analysis data of “pneumonia type” for MUC5B rs35705950 under the TT vs. GG + GT model. Figure S7. Sensitivity analysis (a) and Begg test data (b) for FCGR2A rs1801274 under the allelic T vs. G model. Figure S8. TSA data for FCGR2A rs1801274 under the AG + GG vs. AA model. Figure S9. TSA data for MUC5B rs35705950 under the GT + TT vs. GG model in the overall populations; Figure S10. TSA data for MUC5B rs35705950 under the GT + TT vs. GG model in the Caucasian population; Figure S11. TSA data for MUC5B rs35705950 under the GT + TT vs. GG model in the Asian population. (PDF 1.9 MB) [file 12881_2020_1005_MOESM2_ESM.pdf]

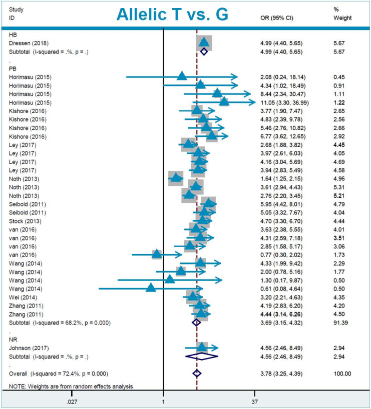
Allelic T vs. G
Study
ID
OR (95% CI)
%
Weight
HB
Dressen (2018) 4.99 (4.40, 5.65) 5.67
Subtotal (I-squared = .%, p = .) 4.99 (4.40, 5.65) 5.67
PB
Horimasu (2015) 2.08 (0.24, 18.14) 0.45
Horimasu (2015) 4.34 (1.02, 18.49) 0.91
Horimasu (2015) 8.44 (2.34, 30.47) 1.11
Horimasu (2015) 11.05 (3.30, 36.99) 1.22
Kishore (2016) 3.77 (1.90, 7.47) 2.65
Kishore (2016) 4.83 (2.39, 9.78) 2.56
Kishore (2016) 5.46 (2.76, 10.82) 2.66
Kishore (2016) 6.77 (3.62, 12.65) 2.92
Ley (2017) 2.68 (1.88, 3.82) 4.45
Ley (2017) 3.97 (2.61, 6.03) 4.05
Ley (2017) 4.16 (3.04, 5.69) 4.69
Ley (2017) 3.94 (2.83, 5.49) 4.58
Noth (2013) 1.64 (1.25, 2.15) 4.96
Noth (2013) 3.61 (2.94, 4.43) 5.31
Noth (2013) 2.76 (2.20, 3.45) 5.21
Seibold (2011) 5.95 (4.42, 8.01) 4.79
Seibold (2011) 5.05 (3.32, 7.67) 4.04
Stock (2013) 4.70 (3.30, 6.70) 4.44
van (2016) 3.63 (2.38, 5.55) 4.01
van (2016) 4.31 (2.59, 7.18) 3.51
van (2016) 2.85 (1.58, 5.17) 3.06
van (2016) 0.77 (0.30, 2.02) 1.73
Wang (2014) 4.33 (1.99, 9.42) 2.29
Wang (2014) 2.00 (0.78, 5.16) 1.77
Wang (2014) 1.30 (0.17, 9.87) 0.50
Wang (2014) 0.61 (0.08, 4.64) 0.50
Wei (2014) 3.20 (2.21, 4.63) 4.35
Zhang (2011) 4.19 (2.83, 6.20) 4.20
Zhang (2011) 4.44 (3.14, 6.26) 4.50
Subtotal (I-squared = 68.2%, p = 0.000) 3.69 (3.15, 4.32) 91.39
NR
Johnson (2017) 4.56 (2.46, 8.49) 2.94
Subtotal (I-squared = .%, p = .) 4.56 (2.46, 8.49) 2.94
Overall (I-squared = 72.4%, p = 0.000) 3.78 (3.25, 4.39) 100.00
NOTE: Weights are from random effects analysis
.027
1
37

Figure S1

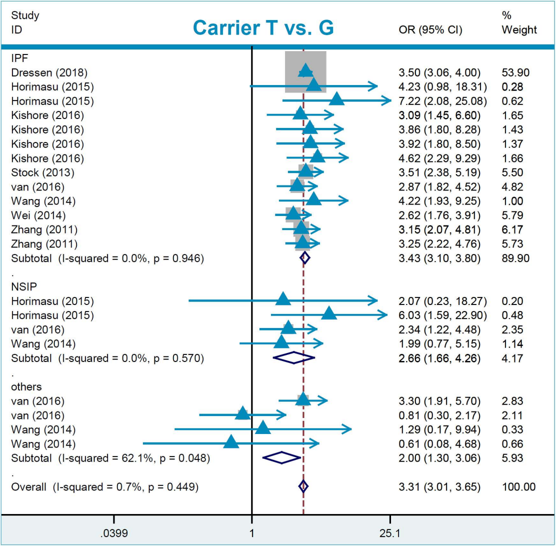
Study
ID
Carrier T vs. G
OR (95% CI)
%
Weight
IPF
Dressen (2018) 3.50 (3.06, 4.00) 53.90
Horimasu (2015) 4.23 (0.98, 18.31) 0.28
Horimasu (2015) 7.22 (2.08, 25.08) 0.62
Kishore (2016) 3.09 (1.45, 6.60) 1.65
Kishore (2016) 3.86 (1.80, 8.28) 1.43
Kishore (2016) 3.92 (1.80, 8.50) 1.37
Kishore (2016) 4.62 (2.29, 9.29) 1.66
Stock (2013) 3.51 (2.38, 5.19) 5.50
van (2016) 2.87 (1.82, 4.52) 4.82
Wang (2014) 4.22 (1.93, 9.25) 1.00
Wei (2014) 2.62 (1.76, 3.91) 5.79
Zhang (2011) 3.15 (2.07, 4.81) 6.17
Zhang (2011) 3.25 (2.22, 4.76) 5.73
Subtotal (I-squared = 0.0%, p = 0.946) 3.43 (3.10, 3.80) 89.90
NSIP
Horimasu (2015) 2.07 (0.23, 18.27) 0.20
Horimasu (2015) 6.03 (1.59, 22.90) 0.48
van (2016) 2.34 (1.22, 4.48) 2.35
Wang (2014) 1.99 (0.77, 5.15) 1.14
Subtotal (I-squared = 0.0%, p = 0.570) 2.66 (1.66, 4.26) 4.17
others
van (2016) 3.30 (1.91, 5.70) 2.83
van (2016) 0.81 (0.30, 2.17) 2.11
Wang (2014) 1.29 (0.17, 9.94) 0.33
Wang (2014) 0.61 (0.08, 4.68) 0.66
Subtotal (I-squared = 62.1%, p = 0.048) 2.00 (1.30, 3.06) 5.93
Overall (I-squared = 0.7%, p = 0.449) 3.31 (3.01, 3.65) 100.00
.0399
1
25.1

Figure S2

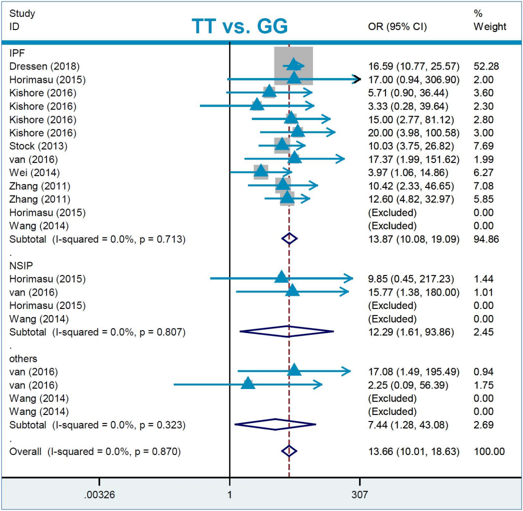
Study
ID
TT vs. GG
OR (95% CI)
%
Weight
IPF
Dressen (2018)
16.59 (10.77, 25.57)
52.28
Horimasu (2015)
17.00 (0.94, 306.90)
2.00
Kishore (2016)
5.71 (0.90, 36.44)
3.60
Kishore (2016)
3.33 (0.28, 39.64)
2.30
Kishore (2016)
15.00 (2.77, 81.12)
2.80
Kishore (2016)
20.00 (3.98, 100.58)
3.00
Stock (2013)
10.03 (3.75, 26.82)
7.69
van (2016)
17.37 (1.99, 151.62)
1.99
Wei (2014)
3.97 (1.06, 14.86)
6.27
Zhang (2011)
10.42 (2.33, 46.65)
7.08
Zhang (2011)
12.60 (4.82, 32.97)
5.85
Horimasu (2015)
(Excluded)
0.00
Wang (2014)
(Excluded)
0.00
Subtotal (I-squared = 0.0%, p = 0.713)
13.87 (10.08, 19.09)
94.86
NSIP
Horimasu (2015)
9.85 (0.45, 217.23)
1.44
van (2016)
15.77 (1.38, 180.00)
1.01
Horimasu (2015)
(Excluded)
0.00
Wang (2014)
(Excluded)
0.00
Subtotal (I-squared = 0.0%, p = 0.807)
12.29 (1.61, 93.86)
2.45
others
van (2016)
17.08 (1.49, 195.49)
0.94
van (2016)
2.25 (0.09, 56.39)
1.75
Wang (2014)
(Excluded)
0.00
Wang (2014)
(Excluded)
0.00
Subtotal (I-squared = 0.0%, p = 0.323)
7.44 (1.28, 43.08)
2.69
Overall (I-squared = 0.0%, p = 0.870)
13.66 (10.01, 18.63)
100.00
.00326
1
307

Figure S3

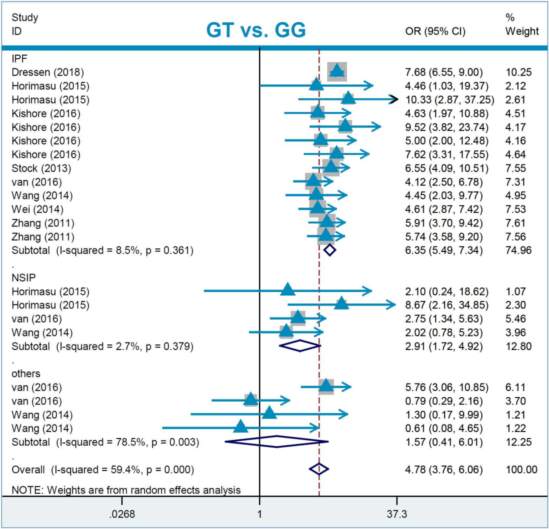
GT vs. GG
Study
ID
OR (95% CI)
%
Weight
IPF
Dressen (2018) 7.68 (6.55, 9.00) 10.25
Horimasu (2015) 4.46 (1.03, 19.37) 2.12
Horimasu (2015) 10.33 (2.87, 37.25) 2.61
Kishore (2016) 4.63 (1.97, 10.88) 4.51
Kishore (2016) 9.52 (3.82, 23.74) 4.17
Kishore (2016) 5.00 (2.00, 12.48) 4.16
Kishore (2016) 7.62 (3.31, 17.55) 4.64
Stock (2013) 6.55 (4.09, 10.51) 7.55
van (2016) 4.12 (2.50, 6.78) 7.31
Wang (2014) 4.45 (2.03, 9.77) 4.95
Wei (2014) 4.61 (2.87, 7.42) 7.53
Zhang (2011) 5.91 (3.70, 9.42) 7.61
Zhang (2011) 5.74 (3.58, 9.20) 7.56
Subtotal (I-squared = 8.5%, p = 0.361) 6.35 (5.49, 7.34) 74.96
NSIP
Horimasu (2015) 2.10 (0.24, 18.62) 1.07
Horimasu (2015) 8.67 (2.16, 34.85) 2.30
van (2016) 2.75 (1.34, 5.63) 5.46
Wang (2014) 2.02 (0.78, 5.23) 3.96
Subtotal (I-squared = 2.7%, p = 0.379) 2.91 (1.72, 4.92) 12.80
others
van (2016) 5.76 (3.06, 10.85) 6.11
van (2016) 0.79 (0.29, 2.16) 3.70
Wang (2014) 1.30 (0.17, 9.99) 1.21
Wang (2014) 0.61 (0.08, 4.65) 1.22
Subtotal (I-squared = 78.5%, p = 0.003) 1.57 (0.41, 6.01) 12.25
Overall (I-squared = 59.4%, p = 0.000) 4.78 (3.76, 6.06) 100.00
NOTE: Weights are from random effects analysis
.0268
1
37.3

Figure S4

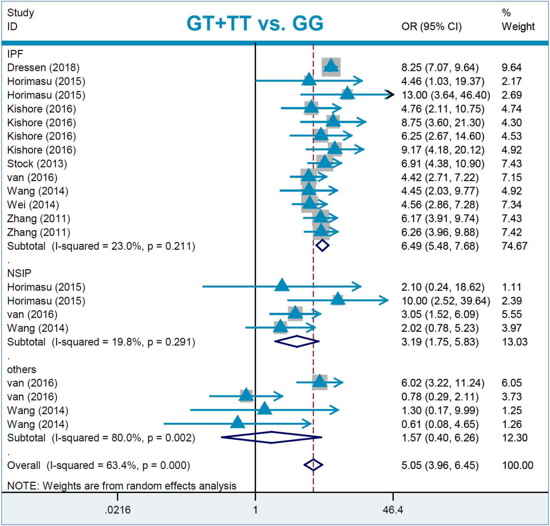
Study
ID
GT+TT vs. GG
OR (95% CI)
%
Weight
IPF
Dressen (2018)
8.25 (7.07, 9.64)
9.64
Horimasu (2015)
4.46 (1.03, 19.37)
2.17
Horimasu (2015)
13.00 (3.64, 46.40)
2.69
Kishore (2016)
4.76 (2.11, 10.75)
4.74
Kishore (2016)
8.75 (3.60, 21.30)
4.30
Kishore (2016)
6.25 (2.67, 14.60)
4.53
Kishore (2016)
9.17 (4.18, 20.12)
4.92
Stock (2013)
6.91 (4.38, 10.90)
7.43
van (2016)
4.42 (2.71, 7.22)
7.15
Wang (2014)
4.45 (2.03, 9.77)
4.92
Wei (2014)
4.56 (2.86, 7.28)
7.34
Zhang (2011)
6.17 (3.91, 9.74)
7.43
Zhang (2011)
6.26 (3.96, 9.88)
7.42
Subtotal (I-squared = 23.0%, p = 0.211)
6.49 (5.48, 7.68)
74.67
NSIP
Horimasu (2015)
2.10 (0.24, 18.62)
1.11
Horimasu (2015)
10.00 (2.52, 39.64)
2.39
van (2016)
3.05 (1.52, 6.09)
5.55
Wang (2014)
2.02 (0.78, 5.23)
3.97
Subtotal (I-squared = 19.8%, p = 0.291)
3.19 (1.75, 5.83)
13.03
others
van (2016)
6.02 (3.22, 11.24)
6.05
van (2016)
0.78 (0.29, 2.11)
3.73
Wang (2014)
1.30 (0.17, 9.99)
1.25
Wang (2014)
0.61 (0.08, 4.65)
1.26
Subtotal (I-squared = 80.0%, p = 0.002)
1.57 (0.40, 6.26)
12.30
Overall (I-squared = 63.4%, p = 0.000)
5.05 (3.96, 6.45)
100.00
NOTE: Weights are from random effects analysis
.0216
1
46.4

Figure S5

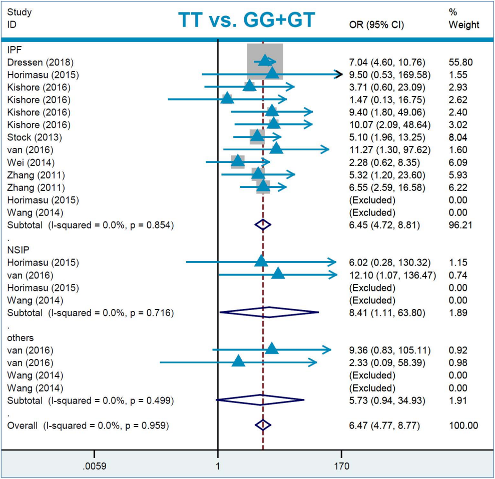
Study
ID
TT vs. GG+GT
OR (95% CI)
%
Weight
IPF
Dressen (2018) 7.04 (4.60, 10.76) 55.80
Horimasu (2015) 9.50 (0.53, 169.58) 1.55
Kishore (2016) 3.71 (0.60, 23.09) 2.93
Kishore (2016) 1.47 (0.13, 16.75) 2.62
Kishore (2016) 9.40 (1.80, 49.06) 2.40
Kishore (2016) 10.07 (2.09, 48.64) 3.02
Stock (2013) 5.10 (1.96, 13.25) 8.04
van (2016) 11.27 (1.30, 97.62) 1.60
Wei (2014) 2.28 (0.62, 8.35) 6.09
Zhang (2011) 5.32 (1.20, 23.60) 5.93
Zhang (2011) 6.55 (2.59, 16.58) 6.22
Horimasu (2015) (Excluded) 0.00
Wang (2014) (Excluded) 0.00
Subtotal (I-squared = 0.0%, p = 0.854) 6.45 (4.72, 8.81) 96.21
NSIP
Horimasu (2015) 6.02 (0.28, 130.32) 1.15
van (2016) 12.10 (1.07, 136.47) 0.74
Horimasu (2015) (Excluded) 0.00
Wang (2014) (Excluded) 0.00
Subtotal (I-squared = 0.0%, p = 0.716) 8.41 (1.11, 63.80) 1.89
others
van (2016) 9.36 (0.83, 105.11) 0.92
van (2016) 2.33 (0.09, 58.39) 0.98
Wang (2014) (Excluded) 0.00
Wang (2014) (Excluded) 0.00
Subtotal (I-squared = 0.0%, p = 0.499) 5.73 (0.94, 34.93) 1.91
Overall (I-squared = 0.0%, p = 0.959) 6.47 (4.77, 8.77) 100.00
.0059
1
170

Figure S6

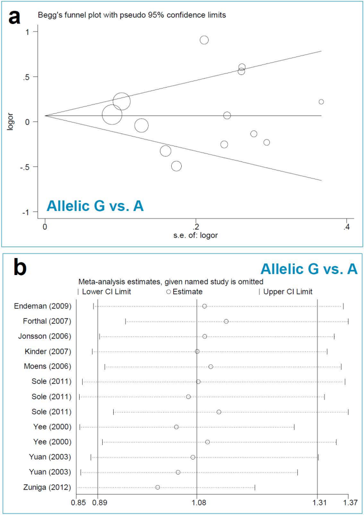

a
Begg's funnel plot with pseudo 95% confidence limits
1
.5
0
-.5
-1
logor
Allelic G vs. A
0
.2
.4
s.e. of: logor
b
Allelic G vs. A
Meta-analysis estimates, given named study is omitted
Lower CI Limit
Estimate
Upper CI Limit
Endeman (2009)
Forthal (2007)
Jonsson (2006)
Kinder (2007)
Moens (2006)
Sole (2011)
Sole (2011)
Sole (2011)
Yee (2000)
Yee (2000)
Yuan (2003)
Yuan (2003)
Zuniga (2012)
0.85
0.89
1.08
1.31
1.37

Figure S7

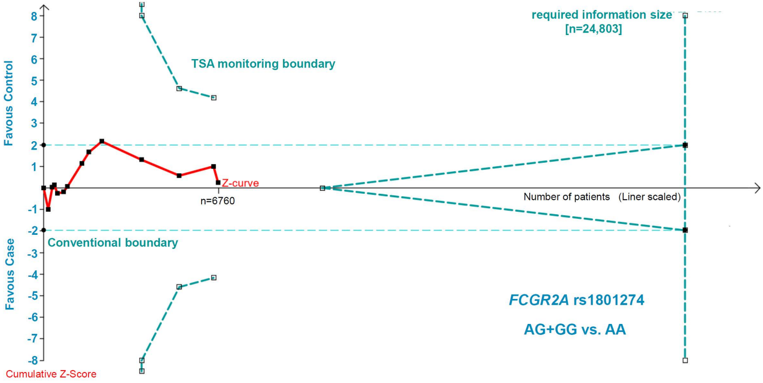
required information size
[n=24,803]
TSA monitoring boundary
Favous Control
Favous Case
Z-curve
n=6760
Number of patients (Liner scaled)
Conventional boundary
FCGR2A rs1801274
AG+GG vs. AA
Cumulative Z-Score
8
7
6
5
4
3
2
1
-1
-2
-3
-4
-5
-6
-7
-8

Figure S8

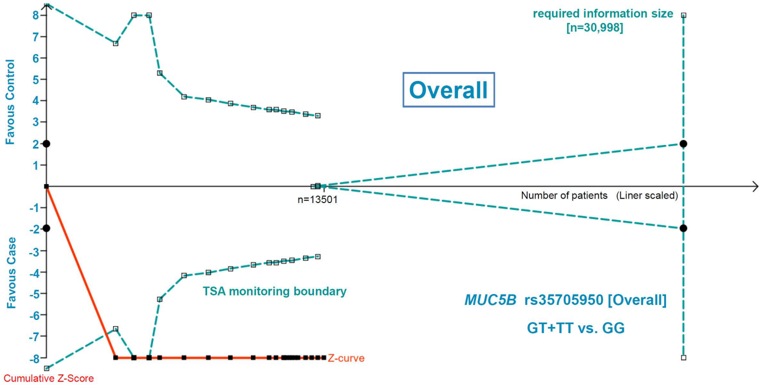

required information size
[n=30,998]
Overall
Favous Control
Favous Case
8
7
6
5
4
3
2
1
-1
-2
-3
-4
-5
-6
-7
-8
n=13501
Number of patients (Liner scaled)
TSA monitoring boundary
MUC5B rs35705950 [Overall]
GT+TT vs. GG
Z-curve
Cumulative Z-Score

Figure S9

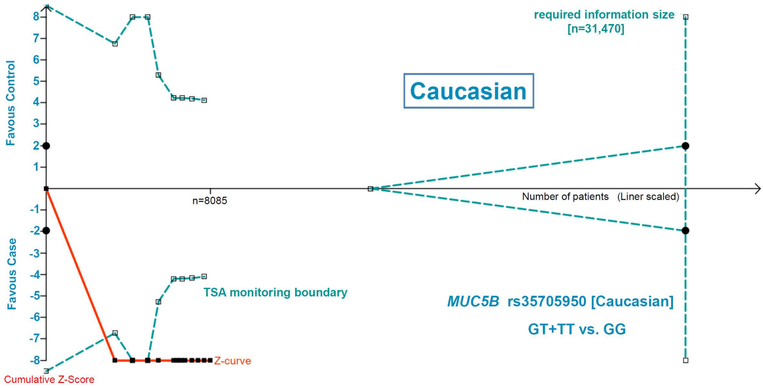

required information size
[n=31,470]
Caucasian
Favous Control
Favous Case
8
7
6
5
4
3
2
1
-1
-2
-3
-4
-5
-6
-7
-8
n=8085
Number of patients (Liner scaled)
TSA monitoring boundary
MUC5B rs35705950 [Caucasian]
GT+TT vs. GG
Z-curve
Cumulative Z-Score

Figure S10

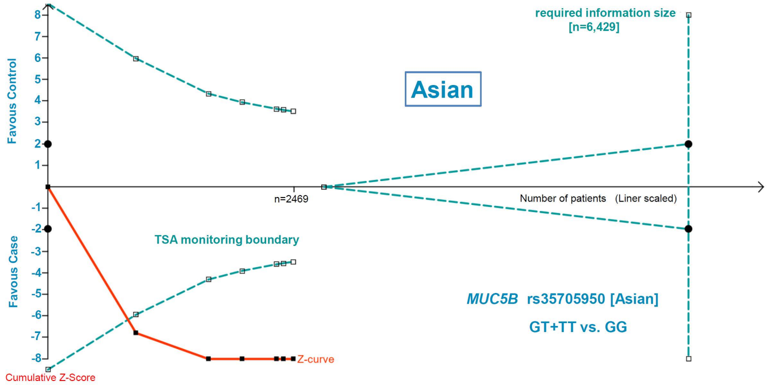

required information size
[n=6,429]
Asian
Favous Control
Favous Case
8
7
6
5
4
3
2
1
-1
-2
-3
-4
-5
-6
-7
-8
n=2469
Number of patients (Liner scaled)
TSA monitoring boundary
MUC5B rs35705950 [Asian]
GT+TT vs. GG
Z-curve
Cumulative Z-Score

Figure S11
